# New Therapies for Sarcoidosis: Molecular and Pathophysiological Basis

**DOI:** 10.3390/ijms27125335

**Published:** 2026-06-12

**Authors:** Fotios Drakopanagiotakis, Ilias Papanikolaou, Theodoros Panou, Elias Gialafos, Nikolaos Kostakis, Konstantinos Chytopoulos, Anastasios Bogiatzis, Paschalis Steiropoulos

**Affiliations:** 1 Department of Pneumonology, Medical School, Democritus University of Thrace, University General Hospital Dragana, 68100 Alexandroupolis, Greecesteiropoulos@yahoo.com (P.S.); 2 Department of Pneumonology, Kerkyra General Hospital, 49100 Corfu, Greece; 3 First Department of Neurology, Medical School, Aeginition Hospital, National and Kapodistrian University of Athens, 11528 Athens, Greece

**Keywords:** sarcoidosis, targeted therapy, JAK-STAT, mTOR, efzofitimod, TNF-α, granuloma, immunotherapy, biomarkers, clinical trials

## Abstract

Sarcoidosis is a multisystem granulomatous disorder of uncertain origin which still presents major therapeutic dilemmas. Longstanding dependence on corticosteroids, while effective for acute inflammation, carries considerable adverse effects over time. Advances in deciphering sarcoidosis pathobiology—including aberrant Janus kinase (JAK)- signal transducer and activator of transcription (STAT) signaling, mechanistic target of rapamycin (mTOR)-driven metabolic shifts, Th1/Th17.1 immune skewing, effector T-cell exhaustion, and granuloma-centered cytokine circuits—have revealed several targets for intervention. The treatment options are rapidly changing: the SARCORT trial showed that low-dose prednisolone is non-inferior to higher prednisolone doses; the pivotal PREDMETH trial validated methotrexate as a feasible first-line steroid-sparing option; efzofitimod, a novel immunomodulator targeting neuropilin-2, produced steroid-reducing effects in Phase IIbut not in Phase III trials; and JAK inhibitors are accumulating evidence across cutaneous and systemic presentations. The 2025 World Association for Sarcoidosis and Other Granulomatoses (WASOG) statement supports a move toward earlier steroid-sparing approaches. This review methodically connects sarcoidosis molecular and pathophysiological mechanisms to new targeted treatments, examines clinical trial evidence, and proposes future directions toward biomarker-driven individualized care.

## 1. Introduction

### 1.1. Epidemiology and Clinical Heterogeneity

Sarcoidosis is a multifaceted, multisystem granulomatous disease of unknown cause that most commonly presents between the ages of 20 and 50. Incidence varies widely by geography and ethnicity, ranging roughly from 1 to 40 cases per 100,000 person-years [1,2]. Sarcoidosis is hallmarked by non-caseating granulomas that can involve nearly any organ, with pulmonary involvement seen in more than 90% of patients and extrapulmonary disease reported in up to 70% [1,3,4,5]. Phenotyping research has delineated discrete clinical clusters that carry different prognoses [3,6,7,8]. These data-driven clusters are distinct from, and extend beyond, the traditional Scadding chest-radiograph stages (0–IV); they integrate the pattern and number of involved organs, demographic factors, and longitudinal disease behavior. Representative clusters include an acute, Löfgren-type presentation with a favorable, often self-limiting course; a predominantly intrathoracic/pulmonary cluster; a multi-organ inflammatory cluster; and a chronic, fibrotic cluster that carries the least favorable prognosis [3,6,7,8]. Eye disease affects approximately 30–60% of patients, cardiac involvement is associated with substantial mortality, and neurosarcoidosis occurs in 5–10% [3,5,6,7,8]. Beyond organ-specific morbidity, patients often suffer from profound fatigue and small fiber neuropathy, which significantly reduce quality of life [5,9]. Recent global burden assessments underscore sarcoidosis’ ongoing and underrecognized impact, particularly among people of African descent [2,10].

### 1.2. Pathophysiological Foundation

**Figure 1:**
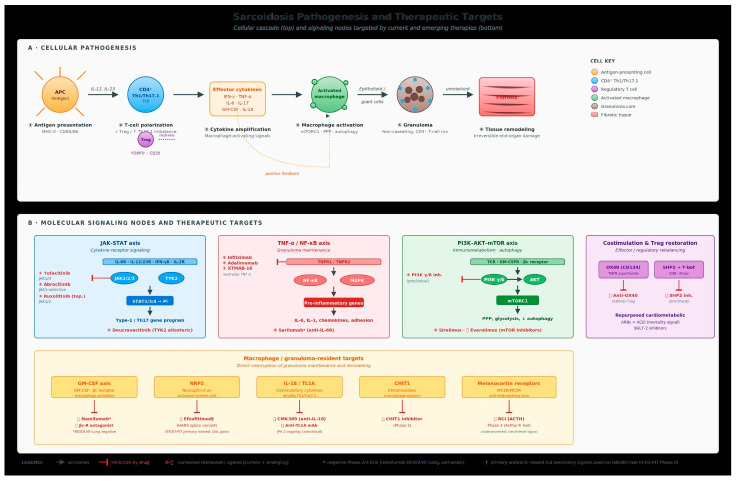
**Sarcoidosis pathogenesis and therapeutic targets: a system-level overview.** This figure provides a whole-pathway map of sarcoidosis and the points at which current and emerging therapies act. (**A**) **Cellular pathogenesis.** The granulomatous cascade proceeds from antigen presentation by antigen-presenting cells (APC; MHC-II, CD80/86) through IL-12/IL-23-driven polarization of CD4^+^ T cells toward a Th1/Th17.1 effector phenotype, with a relative deficiency of regulatory T-cell (Treg) restraint; effector cytokine amplification (IFN-γ, TNF-α, IL-6, IL-17, GM-CSF, and IL-18); macrophage activation with mTORC1-dependent metabolic reprogramming; assembly of the non-caseating granuloma with its CD4^+^ T-cell rim; and, when inflammation is unresolved, progression to fibrosis and irreversible end-organ damage. A positive-feedback loop links effector cytokines back to macrophage activation. (**B**) **Molecular signaling nodes and therapeutic targets.** Four signaling axes implicated in granuloma formation and maintenance are shown—JAK-STAT, TNF-α/NF-κB, PI3K-AKT-mTOR, and costimulation/Treg restoration—together with a macrophage/granuloma-resident target strip (GM-CSF/βc receptor, neuropilin-2 [NRP2], IL-18/TL1A, chitotriosidase [CHIT1], and melanocortin receptors). Numbered red symbols (①–⑳) indicate the molecular site of action of individual agents: ① tofacitinib, ②abrocitinib, ③ topical ruxolitinib, ④deucravacitinib (TYK2, allosteric), ⑤ infliximab, ⑥ adalimumab, ⑦ XTMAB-16, ⑧ sarilumab, ⑨ PI3Kγ/δ inhibitor, ⑩ sirolimus.Circled numbers in Panel (**A**) (①–⑥) denote sequential pathogenic stages and are independent of the agent numbering in Panel (**B**). Arrows denote activation; bar-headed connectors denote pharmacologic inhibition. Asterisks (*) mark agents whose pivotal Phase 2/3 trials were negative (namilumab, RESOLVE-Lung; and sarilumab); the dagger (†) marks efzofitimod, whose Phase 3 EFZO-FIT trial missed its primary endpoint but showed positive secondary signals on patient-reported outcomes. Figure 1 presents the disease-level context; the intracellular mechanisms summarized here as pathway modules are shown at single-cell molecular resolution in Figure 2.

The precise mechanisms initiating sarcoidosis are not fully resolved. Individuals with genetic susceptibility appear to have impaired immune clearance of external triggers, leading to granuloma formation, persistence, and eventual fibrosis [3,11]. The prototypical non-caseating granuloma reflects an organized immune reaction composed of activated macrophages, epithelioid cells, multinucleated giant cells, and a rim of CD4+ T lymphocytes [1,5,12,13,14]. Contemporary pathogenic frameworks stress the interplay of several central processes: a reduced regulatory T cell (Treg)/increased T helper 17.1(Th17.1) balance [15,16,17], exhaustion of effector T cells [12], granulocyte-macrophage colony-stimulating factor (GM-CSF)-driven macrophage activation, mechanistic target of rapamycin complex 1 (mTORC1)-mediated metabolic reprogramming [13,18,19], dysregulated Janus Kinase/signal transducer and activator of transcription (JAK-STAT) signaling [20,21], and expansion of programmed cell death protein 1 (PD-1)+ CD4+ T-cell populations [22]. The notion of an “inflammazone”—shared inflammatory pathways across sarcoidosis and other chronic inflammatory diseases—has expanded conceptual models [23]. Genetic studies have identified susceptibility loci that contribute to disease risk [11,24]. Neutrophil extracellular traps have been implicated in caseating granulomas, highlighting additional innate immune contributions [25]. Mapping these molecular cascades provides the mechanistic basis for the targeted therapies reviewed here (Figure 1).

**Figure 2:**
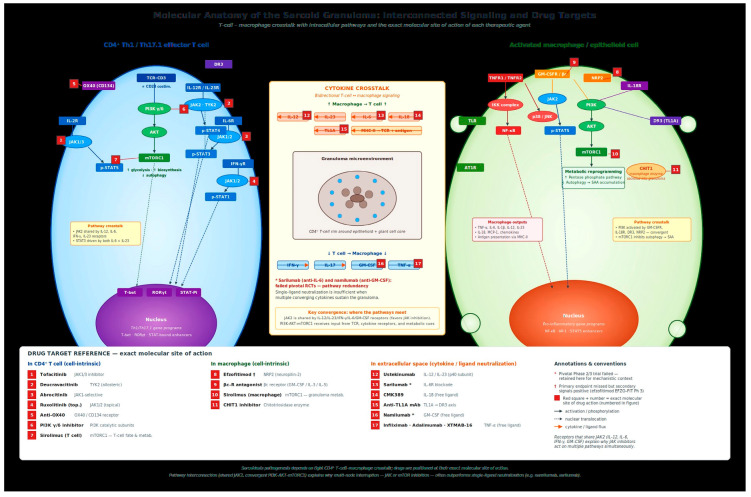
**Molecular anatomy of the sarcoid granuloma: interconnected intracellular signaling and the exact molecular targets of therapy.** Complementing the system-level view of Figure 1, this figure depicts the two principal effector cells of the granuloma—the CD4^+^ Th1/Th17.1 effector T cell (**left**) and the activated macrophage/epithelioid cell (**right**)—at the level of individual receptors, intracellular kinases, and transcription factors and maps each therapeutic agent to its precise molecular site of action. In the T cell, cytokine receptors (IL-12R/IL-23R, IL-6R, IFN-γR, and IL-2R), the T-cell receptor (TCR-CD3/CD28), and the costimulatory receptor OX40 (CD134) signal through paired JAK kinases (JAK1/2/3 and TYK2) to phosphorylate STAT1/3/4/5 and through PI3K-AKT-mTORC1, converging on the T-bet, RORγt, and STAT-dependent gene programs that define the Th1/Th17.1 phenotype. In the macrophage, TNFR1/2, GM-CSFR/βc, NRP2, IL-18R, DR3 (TL1A), Toll-like receptors, and AT1R drive NF-κB, MAPK (p38/JNK), JAK2-STAT5, and PI3K-AKT-mTORC1 signaling, the last promoting pentose-phosphate-pathway flux, reduced autophagy, and serum amyloid A accumulation, alongside secretion of chitotriosidase (CHIT1). The central panel shows the bidirectional cytokine crosstalk that couples the two cells: macrophage-derived IL-12, IL-23, IL-6, IL-18, TL1A, and MHC-II-restricted antigen presentation drive T-cell polarization, while T-cell-derived IFN-γ, IL-17, GM-CSF, and TNF-α reinforce macrophage activation around the epithelioid/giant-cell granuloma core with its CD4^+^ T-cell rim. Numbered red symbols (1–17) indicate the exact molecular target of each agent, grouped by compartment: cell-intrinsic T-cell targets (1 tofacitinib [JAK1/3], 2 deucravacitinib [TYK2], 3 abrocitinib [JAK1], 4 topical ruxolitinib [JAK1/2], 5 anti-OX40, 6 PI3Kγ/δ inhibitor, and 7 sirolimus [mTORC1]); cell-intrinsic macrophage targets (8 efzofitimod [NRP2], 9 βc-receptor antagonist, 10 sirolimus [mTORC1], and 11 CHIT1 inhibitor); and extracellular ligand/receptor neutralization (12 ustekinumab [IL-12/IL-23 p40], 13 sarilumab [IL-6R], 14 CMK389 [IL-18], 15 anti-TL1A, 16 namilumab [GM-CSF], and 17 infliximab/adalimumab/XTMAB-16 [TNF-α]). The figure emphasizes pathway interconnection—JAK2 is shared by the IL-12, IL-23, IFN-γ, IL-6, and GM-CSF receptors, and PI3K-AKT-mTORC1 integrates signals from the TCR, multiple cytokine receptors, and metabolic cues—which provides a mechanistic rationale for why interventions at convergent nodes (JAK or mTOR inhibition) may outperform neutralization of a single cytokine, as suggested by the negative namilumab and sarilumab trials. Arrows denote activation or phosphorylation; dashed arrows denote nuclear translocation; orange arrows denote cytokine/ligand flux. * Negative pivotal trial; † Phase 3 primary endpoint missed with positive secondary signals (efzofitimod).

### 1.3. Unmet Therapeutic Needs

Despite extensive clinical experience, treatment strategies for sarcoidosis remain largely empirical [26,27]. Major unresolved areas include: defining the optimal initial corticosteroid dose; whether corticosteroids can be given as first-line therapy; appropriate treatment duration; therapies that prevent progression to fibrosis; indications for upfront combination regimens; development of sarcoidosis-specific biologics; and shifting primary endpoints toward patient-reported outcomes and steroid-tapering metrics [26,27,28,29]. Analyses of US prescribing patterns from 2016 to 2022 indicate continued usage of corticosteroids [30,31]. The position statement of the 2025 World Association for Sarcoidosis and Other Granulomatoses (WASOG) endorses a change in practice toward earlier steroid-sparing approaches [27]. Three interrelated clinical problems sharpen the rationale for targeted and individualized therapy. First, the response to existing treatment is markedly heterogeneous: many patients enter spontaneous or treatment-induced remission, whereas others develop chronic, relapsing, or progressive fibrotic disease despite adequate immunosuppression, while corticosteroid adherence is itself an additional source of variability [32]. Second, it remains difficult to identify, at presentation, the minority of patients who will progress and therefore stand to benefit most from early intensification, since current clinical and radiographic predictors have only modest discriminative value. Third, there are as yet no validated predictive biomarkers to guide treatment selection. Together these gaps explain why a corticosteroid-centered approach may be regarded as inadequate and motivate the shift toward mechanism-based, biomarker-informed care discussed below [33].

## 2. Molecular Mechanisms and Therapeutic Targets

Mapping the molecular drivers of sarcoidosis is central to rational drug development (Figure 1). The cellular signaling pathways and the specific molecular nodes targeted by current and emerging therapies are summarized in Figure 2.

### 2.1. TNF-α Signaling → Anti-TNF Therapy

Tumor necrosis factor-α (TNF-α) is pivotal in the genesis and maintenance of the sarcoid granuloma, produced mainly by activated macrophages and, to a lesser extent, T lymphocytes within the lesion [1,5]. It orchestrates several non-redundant steps in granuloma assembly: it upregulates endothelial adhesion molecules and chemokines that recruit circulating monocytes and lymphocytes to sites of antigen persistence, enhances macrophage antigen presentation, and amplifies a broader pro-inflammatory cascade—most notably synergizing with interferon-γ (IFN-γ) to reinforce the type-1 effector program that drives epithelioid transformation and giant-cell formation. TNF-α also sustains the chronicity of established granulomas, which is why its neutralization can produce regression of long-standing disease that is otherwise refractory to corticosteroids. Higher TNF-α activity, particularly spontaneous release from alveolar macrophages, associates with more active and progressive disease, and elevated TNF-α has been linked to a poor-prognosis peripheral-blood lymphopenia phenotype that responds poorly to conventional therapy [34,35,36].TNF-α signals through two distinct receptors—tumor necrosis factor receptor 1 (TNFR1, p55), which is widely expressed and couples to both pro-survival and apoptotic programs, and tumor necrosis factor receptor 2 (TNFR2, p75), which is more restricted to immune and endothelial cells—activating downstream nuclear factor kappa B (NF-κB) and mitogen-activated protein kinase (MAPK) cascades that transcriptionally sustain inflammatory gene expression, alongside caspase-dependent apoptotic pathways that shape cell turnover within the granuloma [34,37]. Investigations into infliximab’s effects on pulmonary and circulating immune populations haveshown that TNF-α blockagehas different effects on bronchoalveolar lavage fluid (BALF) and peripheral blood immune cells: it increases the percentage of BALF CD56+ cells with a decrease in peripheral blood, while it does not affect the percentage of natural killer cells [34]. The central pathogenic role of TNF-α is supported by the clinical benefits observed with infliximab and adalimumab, and a comprehensive meta-analysis has synthesized this evidence [29].

### 2.2. Neuropilin-2

A mechanistically distinct approach targets neuropilin-2 (NRP2) with efzofitimod, a first-in-class biologic derived from a naturally occurring, lung-enriched splice variant of histidyl-tRNA synthetase (HARS) fused to an IgG1 Fc domain. The splice variant retains the structurally conserved WHEP domain, which folds into a helix-turn-helix motif that binds selectively to NRP2, a receptor upregulated on activated myeloid cells at sites of inflammation and enriched within sarcoid granulomas. Engagement of NRP2 promotes an anti-inflammatory macrophage program, downregulating pro-inflammatory cytokines including TNF-α, interleukin-6 (IL-6), and monocyte chemoattractant protein-1 (MCP-1) and receptors such as CD14, thereby attenuating inflammation without broad immunosuppression and, in multiple rodent models of interstitial lung disease, reducing immune-cell infiltration and fibrosis [38,39,40].

### 2.3. Janus Kinase/Signal Transducer and Activator of Transcription (JAK/STAT) Pathway → JAK Inhibitors

The Janus kinase–signal transducer and activator of transcription (JAK-STAT) cascade transduces signals from a large set of cytokine receptors implicated in sarcoidosis, including those for IL-2, IL-6, IL-12, IL-23, and—critically—IFN-γ, positioning it as a convergence point downstream of several pathways that are independent lines of evidence that implicate in granuloma formation [20,21].Ligand binding induces receptor dimerization and activation of receptor-associated JAKs (JAK1, JAK2, JAK3, and tyrosine kinase 2 (TYK2)), which phosphorylate STAT transcription factors; phosphorylated STATs dimerize, translocate to the nucleus, and drive the pro-inflammatory transcriptional programs—chiefly STAT1- and STAT4-dependent type-1 responses—that characterize active disease. Because IFN-γ signaling is STAT1-dependent and several granuloma-sustaining cytokines feed into this hub, pharmacologic JAK inhibition can dampen multiple effector arms simultaneously. Damsky and colleagues demonstrated that tofacitinib-mediated inhibition of type-1 immunity produced marked clinical and histologic improvement in longstanding, treatment-refractory sarcoidosis, providing proof-of-concept that the pathway is not merely a correlate but a driver of disease [20].This is consistent with the finding that circulating T cells in sarcoidosis exhibit an aberrantly activated phenotype that correlates with disease outcome and is propagated through JAK-STAT-dependent signaling [12]. Differing selectivity among agents—e.g., tofacitinib (JAK1/3), deucravacitinib (TYK2), and abrocitinib (JAK1)—may permit individualized therapeutic strategies [21,41,42].

### 2.4. IL-12/IL-23 Axis and Th1/Th17.1 Polarization

The IL-12/IL-23 cytokine axis governs the differentiation and stabilization of the pathogenic T-cell populations that define sarcoid immunopathology. IL-12, acting through STAT4, promotes Th1 commitment and IFN-γ production, while IL-23 stabilizes and expands Th17 and the IFN-γ/IL-17 co-producing Th17.1 subset now regarded as a dominant pathogenic effector in sarcoidosis; both cytokines share the p40 subunit, the target of ustekinumab [20,43]. Reciprocal interactions between M2-like macrophages and Th17.1 cells promote progression of early-stage disease, establishing a self-reinforcing loop in which polarized macrophages and effector T cells sustain one another [14]. The balance between regulatory and effector populations appears decisive for disease trajectory: a skewed regulatory T-cell (Treg)/Th17.1 ratio in peripheral blood and bronchoalveolar lavage fluid is associated with disease activity and the fibrotic radiographic phenotype [15,44], and FOXP3^+^ regulatory T cells relate to severity and clinical outcome [16].A greater abundance of functional Treg subsets is associated with a more favorable prognosis, consistent with their role in restraining the granulomatous inflammatory response [15].Therapeutically, this axis is addressable both directly, through anti-p40 (ustekinumab) and related anti-IL-23 strategies, and indirectly, through JAK inhibitors that interrupt IL-12/IL-23 receptor signaling at the level of STAT phosphorylation.

### 2.5. mTOR Signaling → mTOR Inhibitors

The mechanistic target of the rapamycin (mTOR) pathway, and particularly the mTORC1 complex, links immunometabolism to granuloma biology by controlling the anabolic programs that activate macrophages and T cells required for proliferation, differentiation, and effector function [18,19,45,46]. In macrophages, mTORC1-driven metabolic reprogramming—including activation of the pentose phosphate pathway—is essential to generate the biosynthetic precursors and redox cofactors that sustain granulomatous inflammation, and interruption of this metabolic node attenuates granuloma formation experimentally [15]. Dysregulated autophagy together with mTORC1 hyperactivation has been implicated in disease pathogenesis, including in the pulmonary accumulation of serum amyloid A (SAA), a granuloma-associated protein that amplifies local inflammatory signaling and may help perpetuate the lesion [12,18]. Epidemiologic registry data indicate lower sarcoidosis incidence among transplant recipients on mTOR inhibitors compared with calcineurin inhibitors [45]. The relationship is not strictly monotonic, however: high mTOR expression within granulomatous lesions does not uniformly predict clinical course, indicating that mTOR activation is necessary for granuloma maintenance but is, on its own, an insufficient biomarker of progression and likely acts in concert with the metabolic, cytokine, and regulatory-cell determinants described above [19].

### 2.6. GM-CSFand Novel Pathways

Granulocyte-macrophage colony-stimulating factor (GM-CSF) occupies a central position in the myeloid axis of sarcoidosis, driving the differentiation of recruited monocytes into the activated, antigen-presenting macrophages and multinucleated giant cells that constitute the granuloma and sustaining their survival and pro-inflammatory polarization. Because GM-CSF amplifies the very effector population that maintains granulomatous inflammation, its neutralization was an appealing strategy, and it furnished the rationale for the anti-GM-CSF monoclonal antibody namilumab, evaluated in the multinational Phase 2 RESOLVE-Lung trial (NCT05314517) [47].

The renin–angiotensin–aldosterone system (RAAS) is increasingly recognized as a local modulator of granulomatous inflammation rather than merely a systemic vasoregulator: angiotensin-converting enzyme is itself a hallmark product of sarcoid epithelioid cells, and angiotensin II can promote macrophage activation and Th1 polarization within lesions [48,49]. This biology aligns with observational evidence that angiotensin-converting enzyme (ACE) inhibitors (ACEIs) and angiotensin-receptor blockers (ARBs) are associated with decreased and improved survival respectively and health outcomes in sarcoidosis, although the retrospective design of such analyses leaves open the possibility of confounding by indication and warrants prospective confirmation [48].

A series of converging studies has refined the cellular and molecular taxonomy of the disease and nominated additional candidate targets.

C-X-C chemokine receptor type 4 positive (CXCR4+) type 1 innate lymphoid cells help differentiate sarcoidosis from other granulomatous dermatoses, offering both diagnostic discrimination and a clue to chemokine-driven cell trafficking in granuloma assembly [49].

In the cluster of differentiation (CD)8^+^ T-cell compartment, Src-homology-2 domain-containing phosphatase-2 (SHP2) promotes disease severity by inhibiting SKP2-mediated ubiquitination of the T-box transcription factor T-bet (TBET); stabilized T-bet reinforces the type-1 effector program that fuels granulomatous injury, implicating the SHP2–SKP2–TBET axis as a tractable node [50].

Costimulatory and regulatory circuits are likewise dysregulated: blockade of OX40 ((tumor-necrosis-factor-receptor-superfamily costimulatory molecule, CD134), a TNF-receptor–superfamily costimulatory molecule on activated T cells, restores regulatory T-cell (Treg) function and dampens effector responses [51], paralleling the observation that pharmacologic inhibition of phosphoinositide-3-kinase γ/δ rescues Treg function and ameliorates pulmonary granulomas in disease models [17]—together pointing to defective Treg-mediated control as a recurrent and potentially druggable theme. Antagonism of the common beta chain (βc) receptor shared by GM-CSF, IL-3, and IL-5 reduces granuloma formation by simultaneously interrupting inflammatory signaling and correcting the aberrant lipid metabolism that supports activated macrophages [52], an approach that, notably, targets the GM-CSF pathway at the receptor level. On the afferent, antigen-recognition side, newly identified sarcoidosis-associated epitopes augment expression of Major Histocompability Complex (MHC)class II, CD80, and CD86 on antigen-presenting cells and may drive the antigen-specific T- and B-cell responses that initiate and perpetuate disease [53].

## 3. Current Treatment Limitations

### 3.1. Corticosteroid Burden

Corticosteroids remain the cornerstone of sarcoidosis therapy, yet population-level data highlight substantial steroid-associated complications [27,54,55]. U.S. prescribing trends indicate ongoing overuse of systemic steroids [30,31]. A randomized study comparing methylprednisolone pulse therapy versus oral prednisone for sarcoid tubulointerstitial nephritis found no advantage for pulses [56]. Historically, corticosteroid therapy has been linked to disease persistence and chronicity [57]. The 2025 WASOG position paper warns that extended corticosteroid exposure may inflict more harm than the disease itself [27]. Interest has resurfaced in inhaled budesonide as an adjunct for mild pulmonary disease [58].

### 3.2. Conventional Immunosuppressive Agents

Second-line immunosuppressants can spare steroids but show only moderate efficacy, with methotrexate response rates of about 60–70% [1,55,59]. Methotrexate polyglutamate levels may predict therapeutic response [60]. In non-anterior sarcoid uveitis, methotrexate produced outcomes comparable to other conventional disease-modifying antirheumatic drugs (DMARDs) [61], while corticosteroids with or without DMARDs were assessed as initial therapy in non-anterior uveitis [62]. Hydroxychloroquine retains a role in selected mild presentations [63]. Mycophenolate mofetil has shown modest benefit in neurosarcoidosis [64]. The slow onset of action, broad immunosuppressive effects, and toxicity profiles limit the utility of these agents [1,5,26].

## 4. Emerging Therapies: From Molecular Targets to Clinical Evidence

**Figure 3:**
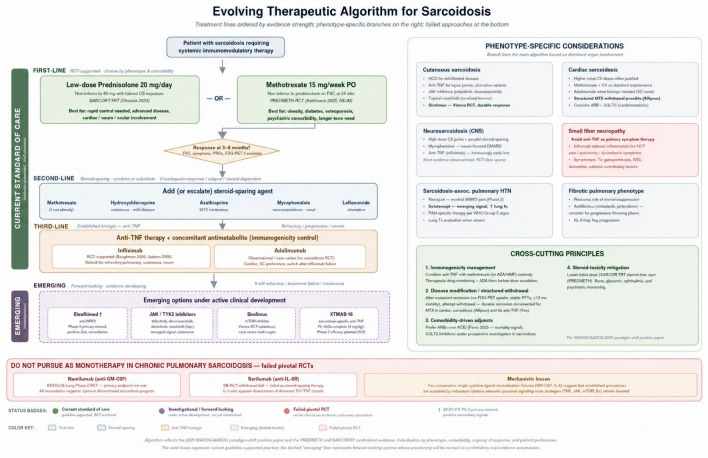
**Evolving therapeutic algorithm for sarcoidosis, with an explicit distinction between current standard of care and investigational options.** This figure translates the biology of Figure 1 and Figure 2 into a practical, evidence-graded treatment framework. The vertical band on the left divides the algorithm into two zones that are reinforced by status badges throughout: a green “current standard of care” zone (guideline-supported, randomized-trial-anchored) spanning the first-, second-, and third-line tiers, and a purple “emerging/investigational” zone for options still under active development. First-line therapy is either low-dose prednisolone (non-inferior to higher doses; SARCORT) or methotrexate (non-inferior to prednisone for forced vital capacity (FVC) at 24 weeks; PREDMETH), chosen by phenotype and comorbidity. Inadequate response, relapse, or steroid dependence prompts second-line steroid-sparing escalation (methotrexate, hydroxychloroquine, azathioprine, mycophenolate, or leflunomide), and refractory or progressive disease prompts third-line anti-TNF therapy with a concomitant antimetabolite (infliximab, randomized-trial-supported; adalimumab, observational). Emerging options (dashed border)—efzofitimod († EFZO-FIT primary endpoint missed, positive secondary signals), JAK/TYK2 inhibitors, sirolimus, and the sarcoidosis-specific anti-TNF XTMAB-16—are shown as forward-looking and explicitly separated from established practice. The right panel summarizes phenotype-specific considerations (cutaneous, cardiac, neurosarcoidosis, sarcoidosis-associated pulmonary hypertension; fibrotic pulmonary disease; and small-fiber neuropathy—the last with an explicit caution that anti-TNF agents do not address neuropathic symptoms) and cross-cutting principles (immunogenicity management, disease modification/structured withdrawal, comorbidity-driven adjuncts, and steroid-toxicity mitigation). The bottom bar demarcates approaches that should not be pursued as monotherapy in chronic pulmonary sarcoidosis because their pivotal randomized trials were negative (namilumab and sarilumab), with an accompanying note on the mechanistic lesson that established granulomas are sustained by redundant cytokine networks. Status badges: green check, current standard of care; purple circle, investigational/forward-looking; red cross, failed pivotal trial. The algorithm reflects the 2025 WASOG/AASOG position paper together with the PREDMETH and SARCORT randomized evidence; treatment should be individualized by phenotype, comorbidity, urgency of response, and patient preference.

The agents below are ordered by the strength of their supporting evidence rather than by mechanistic appeal alone. Three tiers can be distinguished. The first comprises therapies supported by randomized controlled trials: corticosteroid dose optimization (SARCORT); methotrexate as a first-line steroid-sparing option (PREDMETH); anti-TNF therapy—chiefly infliximab—and efzofitimod, which has completed a positive Phase II trial. The second comprises therapies supported mainly by registries, observational cohorts, or small uncontrolled series, such as JAK and mTOR inhibitors, for which much of the current evidence is confined to cutaneous and other selected phenotypes. The third comprises experimental, hypothesis-generating approaches resting on isolated case reports, early-phase signals, or preclinical models, including anti-TL1A strategies, chitotriosidase (CHIT1) inhibition, and several of the additional emerging agents discussed below. This hierarchy is is reflected in the therapeutic algorithm (Figure 3); throughout, we have sought to separate established clinical evidence from preliminary human data and from purely mechanistic rationale.

### 4.1. Optimizing Corticosteroid Regimens: The SARCORT Trial

The SARCORT trial (NCT03265405) was the first randomized controlled study to directly compare a low-dose prednisolone regimen (20 mg/day) with a conventional higher-dose regimen (40 mg/day) in 86 treatment-naïve patients with symptomatic pulmonary sarcoidosis [65]. The trial was designed as a non-inferiority study with a composite primary outcome that included treatment failure, disease progression, and clinically significant relapse over a 12-month tapered course, an endpoint structure that intentionally captured the full spectrum of glucocorticoid-related clinical events rather than any single physiological surrogate. The principal result was that the low-dose regimen was non-inferior to the higher-dose regimen across the composite endpoint: relapse rates were comparable between arms (in the range of 40–45% in both groups), treatment failure was numerically infrequent (approximately 14% with low-dose vs. 9% with high-dose, a difference within the pre-specified non-inferiority margin), and disease progression was observed in fewer than 10% of participants overall [65]. Crucially, FVC and other physiologic endpoints did not differ meaningfully between arms, while the cumulative corticosteroid exposure was, by design, roughly halved in the low-dose group, with a corresponding reduction in glucocorticoid-attributable adverse events, such as hyperglycemia, weight gain, and Cushingoid features [65]. Taken together, these findings provide the first robust randomized evidence that initiating therapy at 20 mg/day is a reasonable default for most patients with symptomatic pulmonary sarcoidosis and represent an important departure from the historically empirical 0.5–1.0 mg/kg/day starting doses derived from older observational practice [65]. The trial does have limitations that bear on generalizability—it was single-center, enrolled patients with relatively newly diagnosed pulmonary-predominant disease, and was not powered to detect differences in less common but clinically important events, such as cardiac or neurologic involvement, where higher initial doses may still be justified—but it has nonetheless become the most influential contemporary evidence informing the move toward lower starting doses now reflected in the 2025 WASOG paradigm-shift position paper on corticosteroid therapy [27].

Real-world data complement this randomized evidence: in a nationwide Japanese cohort, Sawahata et al. characterized the clinical profile of pulmonary sarcoidosis patients started on systemic steroids and showed that initiation, dose, and duration vary substantially with patient and disease factors—including age, radiographic stage, extrapulmonary involvement (notably cardiac), and the presence of symptomatic respiratory impairment at diagnosis—and that long-term steroid exposure remains common in everyday care despite mounting evidence of its cumulative harm [66]. These observations underscore the need for individualized treatment strategies that move beyond a one-size-fits-all corticosteroid algorithm: while SARCORT establishes 20 mg/day as a defensible starting dose for most patients with pulmonary disease, the real-world heterogeneity captured by Sawahata and colleagues reinforces that initial dose, taper trajectory, and the threshold for adding a steroid-sparing agent should be calibrated to the dominant organ involvement, the severity of physiologic impairment, the patient’s comorbidity profile (particularly diabetes, osteoporosis risk, and obesity, where corticosteroid toxicity is amplified), and an explicit, shared assessment of the likelihood and consequences of relapse [27,55]. This individualized framework is precisely the direction signaled by the recent paradigm-shift literature, which argues for earlier integration of corticosteroid-sparing therapy—particularly methotrexate following the PREDMETH/Kahlmann head-to-head data described below [59]—rather than escalating or prolonging corticosteroid exposure as the default response to incomplete remission or relapse [27].

### 4.2. Methotrexate as First-Line Therapy: The PREDMETH Trial

The PREDMETH trial (NCT04314193), reported by Kahlmann and colleagues, was the first multicenter randomized study to directly compare oral prednisone with methotrexate as initial therapy in treatment-naïve patients with symptomatic pulmonary sarcoidosis requiring systemic treatment [59]. The trial was designed as a non-inferiority study with a pre-specified margin of 5% for the between-group difference in change in percent-predicted FVC at 24 weeks, an endpoint chosen because it captures the physiologic response that most directly informs treatment decisions in pulmonary disease and aligns with the outcomes recently prioritized in the international Delphi exercise on pulmonary sarcoidosis trial endpoints [27]. Patients were randomized to a tapered prednisone regimen (starting at 40 mg/day with a protocolized reduction) or to weekly oral methotrexate titrated up to 15 mg/week with mandatory folic acid supplementation and were followed for FVC, symptom burden, quality of life, and adverse-event profile. Methotrexate met the non-inferiority criterion despite a slower early FVC trajectory: prednisone produced a larger and more rapid FVC gain in the first 4–8 weeks—consistent with its faster anti-inflammatory action—but by 24 weeks the between-arm difference narrowed within the pre-specified margin, and longer-term physiologic outcomes were comparable [59]. Methotrexate-treated patients experienced substantially less weight gain, fewer Cushingoid features, and a lower burden of glucocorticoid-attributable metabolic adverse events (hyperglycemia, mood disturbance, sleep disruption), while methotrexate-related toxicities—chiefly gastrointestinal intolerance, modest transaminitis, and mild cytopenias—were generally manageable with dose adjustment and did not lead to disproportionate discontinuation [59]. Patient-reported outcomes, including fatigue and health-related quality of life, also favored the methotrexate arm at later timepoints, a finding of particular relevance given that fatigue and steroid-related morbidity drive much of the long-term disability in sarcoidosis even when objective lung function is preserved.

These results have been widely interpreted as a genuine paradigm shift for first-line treatment of pulmonary sarcoidosis [59,67]. In the accompanying editorial, Baughman and Lower argued that the trial establishes methotrexate as an acceptable steroid-free first-line option for many patients with symptomatic pulmonary disease and that the historical default of prednisone monotherapy—driven by familiarity and rapid onset rather than by direct comparative evidence—should now be reconsidered, particularly for patients in whom corticosteroid-related morbidity is anticipated to be especially burdensome (those with obesity, diabetes, osteoporosis risk, or psychiatric comorbidity) [68]. In this framework methotrexate is no longer reserved as a second-line, steroid-sparing agent but is offered upfront as one of two evidence-based first-line options, with the choice individualized to the patient’s phenotype, comorbidities, urgency of clinical response, and preferences regarding adverse-effect profile [5]. The Delphi exercise on pulmonary sarcoidosis trial endpoints reinforces this individualized framing by recognizing that physiologic, symptom-based, and quality-of-life outcomes all merit weight in defining a successful treatment response and that no single endpoint can capture the multidimensional impact of the disease [27]. Important caveats should be considered. PREDMETH enrolled patients with predominantly pulmonary disease requiring treatment, not the full spectrum of sarcoidosis, and the slower onset of methotrexate’s effect makes it less suitable when rapid control is clinically necessary—for example, in advanced respiratory compromise, sight-threatening uveitis, neurosarcoidosis with active inflammation, or hemodynamically significant cardiac disease, where prednisone (often at higher doses) or combination therapy remains appropriate as first-line treatment [55]. The trial also does not directly address the question of long-term remission and relapse trajectories after treatment withdrawal, which is the dimension most relevant to patients facing a chronic, often relapsing disease [59].

In a prospective cohort study of cardiac sarcoidosis, Alipour and colleagues reported durable remission following methotrexate discontinuation in patients who had achieved sustained disease control, with continued absence of active inflammation on imaging and stable cardiac function during off-treatment follow-up [69]. This finding is clinically important for two reasons. First, it directly counters the long-standing assumption that immunosuppression in cardiac sarcoidosis must be continued indefinitely, raising the possibility of structured withdrawal in carefully selected patients. Second, it parallels the emerging signal across organ systems—including the pulmonary setting—that methotrexate may modify the underlying granulomatous process rather than simply suppress its surface manifestations, an effect more consistent with disease modification than with the symptomatic anti-inflammatory action of corticosteroids alone.

### 4.3. TNF-α Inhibitors

Among biologic therapies, TNF-α inhibitors hold the strongest evidence base in sarcoidosis. The foundational randomized data come from two Phase 2 trials. The Baughman 2006 trial [70] randomized 138 patients with chronic pulmonary sarcoidosis to infliximab (3 or 5 mg/kg) or a placebo over 24 weeks and met its primary endpoint with a mean 2.5% improvement in percent-predicted FVC versus no change with the placebo (*p* = 0.038), although secondary endpoints—quality of life (SGRQ), 6min walk distance, Borg dyspnea score, and lupus pernio response—did not differ significantly from the placebo [70]. The 2.5% FVC change has been criticized as small and of unclear clinical significance, and a key limitation was that corticosteroids were not tapered, leaving the steroid-sparing effect—arguably the most clinically meaningful benefit—unquantified. The companion Judson 2008 randomized controlled trial (RCT) in 92 patients with extrapulmonary sarcoidosis refractory to chronic glucocorticoids showed modest improvement at 24 weeks that was not maintained over a 24-month follow-up, again without a structured steroid taper [71]. The Bechman meta-analysis confirms a modest but consistent FVC improvement and reliable steroid-sparing effect for anti-TNF therapy across pulmonary and extrapulmonary disease, with much weaker evidence for most other targeted agents [29].

Efficacy distributes unevenly across phenotypes. In refractory pulmonary sarcoidosis, infliximab carries the most extensive evidence, with real-world multicenter analyses confirming FVC and steroid-sparing benefits outside the trial setting [72]; response is most predictable in patients with active alveolitis on BAL or persistent ^18^F-FDG uptake on PET, and mechanistic data show that infliximab remodels multiple immune compartments—alveolar macrophages, natural killer (NK) cells, CD56^+^ T cells, and B cells—rather than acting through a single lineage [34]. In extrapulmonary disease, the Judson 2008 RCT and subsequent observational data suggest patients may derive the greatest sustained benefit from long-term anti-TNF therapy, consistent with the clinical impression that skin, eye, CNS, and cardiac granulomas respond more visibly than the lung [71]. In cutaneous sarcoidosis, including lupus pernio, ulcerative variants, and recalcitrant systemic forms, infliximab is among the most effective options; the negative lupus pernio result at week 24 in the RCT by Baughman et al. reflects endpoint limitations and slow cutaneous granuloma resolution rather than true inefficacy [70]. In cardiac sarcoidosis, adalimumab is a reasonable alternative offering subcutaneous administration and reported efficacy in case series [73]. In neurosarcoidosis, anti-TNF therapy is increasingly first-line for parenchymal and meningeal disease despite sparse direct trial data [29]. In sarcoidosis-associated small fiber neuropathy, however, infliximab reduces inflammatory activity but has no effect on neuropathy symptoms, indicating that pain and autonomic dysfunction in this phenotype are not TNF-driven and require a different strategy [9]—a clinically important negative finding that should redirect therapy in such patients.

Adalimumab, a fully human antibody, has lower intrinsic immunogenicity than chimeric infliximab and is administered subcutaneously, making it attractive for both primary and switch indications. In a Dutch cohort of 18 patients switched from infliximab to adalimumab for intolerance or progression, 7 improved their FVC, 6 stabilized, and 5 deteriorated, with one lupus-like syndrome, three severe infections, and seven mild infections [74]. Therefore, adalimumab might be a reasonable salvage after infliximab failure but is generally less pronounced and slower in pulmonary onset.

Immunogenicity is a limitation of infliximab: anti-drug antibodies cause loss of response and infusion reactions, and therapeutic drug monitoring combined with concomitant methotrexate (or, if intolerant, azathioprine or mycophenolate) may preserve efficacy [75]. Medication-free remission has been described after planned anti-TNF withdrawal in selected patients [76].

XTMAB-16, a chimeric monoclonal antibody developed specifically for sarcoidosis to address the immunogenicity and pharmacokinetic limitations of repurposed biologics, has progressed through Phase 1 and is in a seamless Phase 1b/2 trial in pulmonary sarcoidosis (NCT05890729) with an open-label extension (NCT06169397), structured around a corticosteroid-tapering endpoint and carrying orphan drug designation in both the U.S. and EU [77]; if positive, it would be the first sarcoidosis-specific anti-TNF agent.

### 4.4. Efzofitimod: Targeting Neuropilin-2

Efzofitimod [ATYR,1923] is an IV biologic derived from a histidyl-tRNA synthetase (HARS) splice variant that binds NRP2, designed to modulate early granulomatous inflammation [38,39,40]. Exposure-response modeling indicates dose-dependent effects [78]. In a Phase II double-blind randomized trial assessing 1, 3, and 5 mg/kg every 4 weeks, the agent was well tolerated without immunogenicity; the 5 mg/kg dose enabled steroid tapering without relapse, produced a 2.5% FVC gain, and achieved quality-of-life improvements exceeding the minimal clinically important difference (MCID) [39,79].In late 2025, results of a Phase III trial called the EFZO-FIT trial were reported in the European Respiratory Society (ERS) congress: the primary endpoint changed from the baseline in mean daily oral corticosteroid dose at week 48. Inclusion criteria were the diagnosis of pulmonary sarcoidosis made more than six months prior to recruitment, a stable oral corticosteroid dose between 7.5 mg and 25 mg daily, with one immunosuppressant agent allowed. There was a 1:1:1 randomization with two different doses of Efzofitimod(5 mg/kg and 3 mg/kg) or a placebo. Two hundred and sixty-eight patients were randomized, making it the largest sarcoidosis study. Although the primary endpoint was not met, since there was not statistically significant difference in corticosteroid reduction between the drug and placebo, there was a significant positive effect of efzofitimod in several secondary endpoints examining quality-of-life parameters, such as the King’s Sarcoidosis Questionnaire score and the Fatigue Assessment Scale [80]. Despite failing to achieve the primary endpoint, these results are promising, and further evaluation is needed, making efzofitimodan advanced biologic developed specifically for sarcoidosis.

### 4.5. JAK Inhibitors

JAK inhibitors directly target the central JAK-STAT signaling hub through which IL-2, IL-6, IL-12, IL-23, and IFN-γ—cytokines independently implicated in granulomatous inflammation—converge to drive the Th1/Th17.1 effector programs that characterize chronic sarcoidosis [20,21]. The current evidence base for JAK inhibition in sarcoidosis is dominated by cutaneous disease and by small, largely uncontrolled case series and individual reports. No randomized controlled trial of a JAK inhibitor in sarcoidosis has yet been completed, and the class therefore remains investigational despite encouraging early signals; extrapolation to pulmonary, cardiac, and neurological phenotypes—where granuloma biology, drug penetration, and the consequences of inadequate disease control all differ from skin—should be made with caution.

The most influential clinical-translational study to date is the open-label experience of Damsky and colleagues in ten patients with cutaneous sarcoidosis (several with extracutaneous involvement), in which tofacitinib (JAK1/3) produced marked clinical improvement that was accompanied by transcriptomic suppression of type-1 immunity in lesional skin, providing rare mechanism-of-action confirmation in human disease rather than relying on phenotype alone [20]. A subsequent systematic review combining case-series data has documented responses to JAK inhibition across multiple sarcoidosis manifestations, including cutaneous, pulmonary, articular, and lymphadenopathic disease, although the underlying studies remain small and heterogeneous, with variable concomitant therapy, inconsistent outcome measures, and short follow-up [21]. Real-world drug-survival data from a Danish registry of refractory sarcoidosis patients showed favorable persistence on tofacitinib (and abatacept), including in patients who had previously failed TNF inhibitors—supporting the practical role of JAK inhibition as a salvage option in refractory disease and reinforcing the impression that immunogenicity and loss of response to anti-TNF therapy are clinically meaningful drivers of treatment switching in sarcoidosis [81]. Beyond pan-JAK inhibition, the differingselectivity profiles of newer agents may permit more individualized therapeutic strategies and partially separate efficacy from class toxicities such as cytopenias, infection, and thrombotic risk. Deucravacitinib, a selective TYK2 inhibitor that uniquely targets the regulatory domain of the kinase rather than its ATP-binding site, has produced resolution of cutaneous sarcoidosis in case reports, and its mechanism—preferential interruption of IL-12, IL-23, and type I interferon signaling without affecting JAK1/2/3—is conceptually well-matched to the Th1/Th17.1 axis in sarcoidosis [81]. Abrocitinib, a JAK1-selective inhibitor, has been effective in tattoo-associated cutaneous sarcoidosis, a phenotype with a particularly characteristic antigen-driven pathogenesis that may make it especially responsive to upstream cytokine-pathway interruption [41]. Topical ruxolitinib has been used for cutaneous disease as a non-systemic option with limited systemic exposure, although the depth of granuloma penetration achievable with topical therapy is unclear, and the durability of response is uncertain [82]. Controlled trials are needed across sarcoidosis phenotypes.

### 4.6. Ustekinumab and the IL-12/IL-23 Axis

Because IL-12- and IL-23-driven Th1/Th17.1 polarization is central to granuloma formation, the anti-IL-12/IL-23 p40 monoclonal antibody ustekinumab was an attractive candidate in sarcoidosis. This rationale was tested in a randomized, double-blind, placebo-controlled Phase 2 trial that evaluated ustekinumab and the anti-TNF-α antibody golimumab against a placebo in patients with chronic pulmonary and/or cutaneous sarcoidosis, with corticosteroid tapering between weeks 16 and 28 [83]. The trial was negative for its primary endpoint: at week 16, neither ustekinumab (ΔFVC −0.15%, *p* = 0.13) nor golimumab (ΔFVC +1.15%, *p* = 0.54) improved percent-predicted FVC relative to the placebo (+2.02%), and none of the major secondary pulmonary endpoints—week-28 FVC, six-minute walk distance, or St George’s Respiratory Questionnaire—were met by either agent. The only positive signal was a nonsignificant numerical trend toward a greater Skin Physician Global Assessment response with golimumab (53% versus 30% for the placebo), suggesting possible, unconfirmed activity in cutaneous disease; ustekinumab showed no such trend. Both biologics were well tolerated, with comparable serious-adverse-event rates [83]. Notably, the only other substantive ustekinumab-in-sarcoidosis literature consists of paradoxical case reports in which ustekinumab, given for psoriasis, induced sarcoid-like granulomatous reactions, further undermining any rationale for its therapeutic use [84,85].

### 4.7. mTOR Inhibitors

Epidemiologic registry data first signaled clinical relevance by showing a lower incidence of new-onset sarcoidosis among solid-organ transplant recipients maintained on mTOR inhibitors compared with those on calcineurin inhibitors, suggesting that mTOR activity contributes to disease initiation in susceptible individuals rather than being merely a downstream correlate of established inflammation [45]. Case series report multi-organ improvement—including pulmonary, cutaneous, cardiac, and constitutional features—in selected refractory patients [46]. The most rigorous clinical evidence to date comes from a single-centerrandomized trial at the Medical University of Vienna in 16 patients with persistent, glucocorticoid-refractory cutaneous sarcoidosis: topical sirolimus showed no benefit, but systemic sirolimus produced clinical improvement in 70% (7/10) of treated patients, with a median Cutaneous Sarcoidosis Activity and Morphology Index (CSAMI) reduction of −7.0 points (*p* = 0.018) and complete resolution in 3 responders; remarkably, the effect was sustained for up to two years after the 4-month treatment course concluded, suggesting that short-course mTOR inhibition may exert a disease-modifying rather than purely suppressive effect on granuloma biology [86]. This durability is conceptually distinct from the pattern seen with corticosteroids and most other immunosuppressants in sarcoidosis, where withdrawal is typically followed by relapse. Overall, the evidence for mTOR inhibition in sarcoidosis remains preliminary—resting on epidemiological associations, uncontrolled case series, and a small single-center cutaneous trial—and adequately powered controlled studies in extracutaneous disease are still lacking. The toxicity profile of systemic sirolimus—including mucositis, dyslipidemia, cytopenias, proteinuria, and an idiosyncratic risk of non-infectious pneumonitis that requires particular vigilance in a pulmonary-sarcoidosis population—also imposes a meaningful constraint on broader deployment and underscores the need for careful patient selection.

### 4.8. Anti-GM-CSF Therapy: Namilumab

Because GM-CSF amplifies the very effector population that maintains granulomatous inflammation, its neutralization was an appealing strategy, and it furnished the rationale for the anti-GM-CSF monoclonal antibody namilumab, evaluated in the multinational Phase 2 RESOLVE-Lung trial (NCT05314517) [47]. The trial enrolled patients with chronic active pulmonary sarcoidosis requiring ongoing immunosuppression and was designed around a corticosteroid-tapering primary endpoint with rescue events during the double-blind period as the failure criterion—an endpoint architecture closely aligned with what the Delphi consensus on pulmonary sarcoidosis trial endpoints would subsequently endorse as clinically meaningful [28]. The trial did not meet its primary endpoint [47]. Namilumab failed to reduce the proportion of subjects experiencing a rescue event during the double-blind period, and pre-specified secondary endpoints—change in percent-predicted FVC, corticosteroid-tapering success, and the King’s Sarcoidosis Questionnaire—likewise showed no treatment benefit, leading to discontinuation of the sarcoidosis development program [47]. This outcome is informative on several levels. Clinically, it tempers enthusiasm for GM-CSF blockade as a stand-alone strategy in chronic pulmonary disease and demonstrates that mechanistic plausibility, even when supported by strong preclinical and translational rationale, does not predict clinical success in established granulomatous inflammation. Mechanistically, it raises the possibility that in mature, chronically active granulomas, GM-CSF signaling is redundant with—or downstream of—other macrophage-activating pathways (notably IL-6, IL-15, IFN-γ, and TNF-α), so that single-ligand neutralization upstream cannot interrupt the self-reinforcing network of cytokines, metabolic reprogramming, and matrix-derived signals that maintains the lesion. Population factors may also have contributed: RESOLVE-Lung enrolled patients with long-established, treatment-refractory chronic disease, and the same intervention applied earlier in the disease course—before structural remodeling and macrophage transcriptional commitment are entrenched—might in principle yield different results.

### 4.9. Additional Emerging Agents

The agents grouped here span widely differing stages of development—from retrospective clinical analyses to purely preclinical models—and are listed in approximately descending order of evidence maturity; most should be regarded as exploratory. Sarilumab, an IL-6 receptor blocker with established efficacy in rheumatoid arthritis and giant cell arteritis, was tested in a double-blind, placebo-controlled, randomized withdrawal trial in glucocorticoid-dependent sarcoidosis and failed to demonstrate a steroid-sparing effect [87].

In sarcoidosis-associated pulmonary hypertension—a high-mortality complication for which dedicated trial evidence remains scarce—sotatercept, an activin signaling inhibitor that has transformed treatment of pulmonary arterial hypertension, has been associated with improved lung function in a recent report [88], while riociguat, a soluble guanylate cyclase stimulator, delivered only modest gains in a one-year double-blind, placebo-controlled trial of sixteen patients with sarcoidosis-associated pulmonary hypertension [89]. Among repurposed and older agents with clinical signals, phosphodiesterase (PDE)-4 inhibitors—such as roflumilast—have shown potential as steroid-sparing options in retrospective single-center analyses, with response across cutaneous, pulmonary, and constitutional manifestations and a favorable safety profile relative to systemic immunosuppression [90]. Repository corticotropin injection (RCI), an older adrenocorticotropic hormone (ACTH)-based therapy, demonstrated modest results in a Phase IV trial [91]. The study was terminated early due to low enrollment during the COVID-19 pandemic (n = 55 randomized; RCI n = 27, placebo n = 28), which precluded formal statistical hypothesis testing, but descriptive analyses showed favorable trends for RCI across multiple endpoints: a greater mean improvement in the novel Sarcoidosis Treatment Score (STS) at week 24 (1.4 vs. 0.7) and at week 48 (1.8 in continued RCI vs. 0.9 in placebo-to-RCI switchers), more glucocorticoid discontinuations in the RCI arm at week 24, and a similar pattern across pulmonary function and patient-reported outcomes, with no new safety signals [86]. Beyond the efficacy signal—which remains hypothesis-generating rather than confirmatory—the trial made a methodologically important contribution by validating the STS and other composite endpoints for use in future, adequately powered sarcoidosis trials [86]. RCI thus retains a niche role in patients who cannot tolerate or have failed standard immunosuppression, though prospective confirmatory evidence is still lacking [91].

In a multicenter analysis of the TriNetX research network, sarcoidosis patients prescribed ACEIs experienced increased 5-year mortality compared with those prescribed ARBs, along with worse cardiac and respiratory outcomes and higher sepsis rates, suggesting that ACEIs and ARBs may have divergent effects on disease course despite acting on the same pathway [48]. Sodium-glucose cotransporter 2 (SGLT-2) inhibitors are being explored for possible mortality benefits. SGLT2 inhibitors modulate intrarenal RAAS activity; attenuate systemic and tissue inflammation through reductions in oxidative stress, NLR family pyrin domain containing 3 (NLRP3) inflammasome signaling, and pro-inflammatory cytokine production; and exert cardio-renal protective effects that have transformed the treatment of heart failure and chronic kidney disease—a mechanistic profile that could plausibly intersect with the inflammatory and end-organ pathways relevant to sarcoidosis. A 2026 repurposing analysis proposed SGLT2 inhibition as a mortality-reduction strategy in sarcoidosis on this basis [92]. CHIT1 inhibition is a novel strategy currently in Phase II testing as a potential first-line therapy [93]. Anti- TNF-like ligand 1A (TL1A) antibodies reduce granuloma formation via phosphoinositide 3-kinase (PI3K)/protein kinase B (AKT) pathway modulation in preclinical work [94].

CMK389, a fully human anti-IL-18 monoclonal antibody, was evaluated in a quadruple-blind, randomized, placebo-controlled Phase 2 proof-of-concept trial in chronic pulmonary sarcoidosis (NCT04064242, n = 62; 10 mg/kg IV every 4 weeks for four doses). Despite a strong rationale—IL-18 synergizes with IL-12 to amplify IFN-γ production and is elevated in the serum and bronchoalveolar lavage fluid of patients with pulmonary sarcoidosis—the trial was negative: the change in percent-predicted FVC at week 16 numerically favored the placebo (−0.48 vs. +1.02), with a Bayesian treatment difference of −1.49 (80% credible interval −3.56 to 0.60). The program was subsequently discontinued [95].

Emerging targeted therapies for sarcoidosis with the grade of evidence are shown it Table 1.

## 5. Clinical Trial Landscape

### 5.1. Completed and Ongoing Trials

The portfolio of clinical trials in sarcoidosis has grown substantially (Table 2). Landmark studies include SARCORT [65], PREDMETH [59], the Phase III efzofitimod trial [38,39,80], and RESOLVE-Lung [96]. Bechman and colleagues have summarized the evidence for biologic and targeted synthetic therapies [29].Negative results, read together with the failed namilumab (anti-GM-CSF) RESOLVE-Lung trial, underscore that biological plausibility alone does not guarantee clinical benefit: the IL-6 axis—and, in that setting, GM-CSF signaling—appears less central to established granulomatous inflammation than the upstream Th1/Th17.1 and TNF-driven circuits, a distinction that could inform the prioritization of future targets.

### 5.2. Advances in Trial Design

Therapeutic trials in sarcoidosis have long been constrained by the heterogeneity of organ involvement, a shortage of validated activity biomarkers, and persistent recruitment challenges. The recent Delphi consensus on pulmonary sarcoidosis trial endpoints directly addressed these obstacles, prioritizing corticosteroid tapering and patient-reported outcomes as clinically meaningful measures of treatment success [28]—an endpoint framework already reflected in the design of contemporary biologic trials such as RESOLVE-Lung and EFZO-FIT. Looking forward, trial efficiency is likely to be improved by adaptive designs and by biomarker-enriched enrollment strategies that concentrate study populations among patients most likely to respond, complemented by emerging molecular tools—including extracellular-vesicle signatures and urinary metabolomic profiling—that may eventually enable both patient stratification and objective monitoring of response.

## 6. Future Directions

### 6.1. Personalized Medicine and Biomarkers

Sarcoidosis treatment efficacywillprobably depend on matching treatments to molecular patient profiles [97]. Targeted proteomic analysis of extracellular vesicles has yielded biomarkers predictive of response [98], such as lower serpin C1 levels predicting methotrexate responsiveness [99]. Biomarker strategies have evolved from ACE and sIL-2R toward integrated multi-omic platforms [100,101]. Combined transcriptomic and metabolomic profiling exposes immune–metabolic derangements [102]. Elevated circulating PD-1+ CD4+ memory T cells have been linked to favorable prednisone responses [12,22]. Mendelian randomization has investigated short telomere length as arisk factor for sarcoidosis [103]. Machine learning approaches may enable predictive therapeutic selection [104]. Beyond molecular signatures, disease expression—and, plausibly, therapeutic response—is shaped by host factors that future precision-medicine frameworks will need to incorporate. Sex and gender influence the age of onset, the pattern of organ involvement, and the quality-of-life burden of sarcoidosis [105], while ancestry contributes to substantial variation in incidence, phenotype, and outcome, with the highest burden among people of African descent [106]. Distinct inflammatory phenotypes—reflecting, for example, the balance between Th1 and Th17.1 responses and the polarization of macrophages—are increasingly recognized and may underlie differential treatment responsiveness [3]. Within the biomarker space, markers associated with a fibrotic interstitial-lung-disease phenotype deserve particular attention: serum Krebs von den Lungen-6 (KL-6), a MUC1-derived glycoprotein released by injured type II pneumocytes, is elevated in fibrotic pulmonary involvement and may have translational value for flagging progressive fibrotic disease in pulmonary sarcoidosis, although assay standardization remains a practical consideration [107,108]. Integrating these demographic, phenotypic, and biomarker dimensions will be essential if treatment selection becomesindividualized.

### 6.2. Combination Therapies and Novel Targets

Strategically combining agents that target complementary pathogenic nodes is an attractive strategy [5,27]. The role of antifibrotic drugs (pirfenidone andnintedanib) for the treatment of pulmonary sarcoidosis remains insufficiently defined despite clinical need [5,27]. Longitudinal lung function trajectories may help guide therapeutic choices [109]. Clinical and imaging predictors of progression have been characterized [3]. Emerging pipeline candidates include anti-TL1A [94], OX40 inhibitors [51], CHIT1 inhibitors [93], SGLT-2 inhibitors [92], βc receptor antagonists [52], and PI3K γ/δ inhibitors [17].

### 6.3. Disease Modification

Treatments that alter natural history and induce sustained remission remain the ultimate objective [26,27,97]. Durable medication-free remission following anti-TNF cessation has been observed [76], as has remission after methotrexate withdrawal in cardiac sarcoidosis [69]. Data encompassing diverse populations will be important to ensure global applicability [67,103]. Incorporating mechanistic discoveries into precision-medicine frameworks holds promise to fundamentally improve outcomes. Identifying patients at high risk for progressive disease and matching them to therapies that target the responsible pathogenetic mechanisms is central to altering sarcoidosis’s trajectory.

## 7. Conclusions

The treatment landscape for sarcoidosis is in transition, propelled by an expanding mechanistic understanding and a growing armamentarium of targeted agents. Convergent molecular insights—JAK-STAT perturbation; mTOR-driven metabolic reprogramming; Th1/Th17.1 imbalance; TNF-α-dependent granuloma maintenance; and newer pathways, such as NRP2 and GM-CSF signaling—have translated into a varied pipeline of therapeutic candidates (Figure 1 and Figure 2; Table 1). Clinically, practice has been influenced by notable developments: SARCORT supporting lower steroid starting doses [65]; PREDMETH validating methotrexate as a first-line option [59]; efzofitimod emerging asa leading sarcoidosis-targeted biologic, despite failing to meet the primary endpoint of oral corticosteroid dose reduction in a Phase III trial [39,80]; and the WASOG 2025 recommendation favoring earlier steroid-sparing approaches [27]. JAK inhibitors, anti-TNF agents, and mTOR inhibitors add to the therapeutic repertoire (Figure 3; Table 1 and Table 2). The precise sequencing and optimal placement of these therapies will need clarification through further study. The future will likely center on biomarker-directed individualized therapy, rational combination regimens, endotyping, and true disease modification. As emerging strategies undergo rigorous evaluation, there is justified optimism that sarcoidosis care will be transformed through more precise, mechanism-driven algorithms [27,33,55,97].

## Figures and Tables

**Table 1:** Emerging targeted therapies for sarcoidosis.

Agent	Drug Class	Molecular Target	Key Evidence	Evidence Level/Stage	Refs
**TIER 1 · Established by RCT-level evidence**					
Infliximab	Anti-TNF-α (chimeric mAb)	TNF-α	Baughman 2006 Phase 2 RCT (n = 138): +2.5% FVC vs. placebo (*p* = 0.038); SGRQ, 6MWT, dyspnea, lupus pernio not significant. Judson 2008 Phase 2 RCT (n = 92, extrapulmonary): modest improvement at 24 wks, not maintained. Real-world multicenter data confirm FVC and steroid-sparing benefit. Greater benefit in more severe diseases (posthoc).	Established (Phase 2 RCT + RWE)	[29,34,70,71,72]
Efzofitimod †	HARS splice variant-Fc fusion	Neuropilin-2 (NRP2) on activated myeloid cells	Phase 2 RCT (Culver 2023, n = 37): well-tolerated, no immunogenicity; 5 mg/kg enabled steroid taper without relapse; +2.5% FVC; KSQ above MCID. Phase 3 EFZO-FIT (n = 268): primary OCS-taper endpoint not met (*p* = 0.33); positive nominal secondary signals on KSQ and Fatigue Assessment Scale.	Established (Ph 2+)/Ph 3 missed primary	[38,39,40,79,80]
**TIER 2 · Phase 2 or substantive mechanistic clinical evidence**					
Adalimumab	Anti-TNF-α (human mAb)	TNF-α	No completed Phase 2/3 RCT in sarcoidosis. Evidence is observational and case-based: Crommelin 2016 switch cohort (n = 18)—7 improved FVC, 6 stable, 5 deteriorated after infliximab failure; Sweis 2022 cardiac sarcoidosis case series; SC route, lower immunogenicity, and slower onset than infliximab. Class-level RCT evidence (Bechman meta-analysis) is largely driven by infliximab.	Substantive clinical/Case series	[29,73,74]
Tofacitinib	JAK1/3 inhibitor	JAK–STAT (Th1/IFN-γ program)	Damsky 2022 open-label clinical-translational study (n = 10, cutaneous): marked clinical improvement with transcriptomic confirmation of type-1 immunity suppression. Multi-organ responses in subsequent case series. Danish registry: favorable drug survival, including after anti-TNF failure.	Emerging—Phase 1–2/Real-world	[20,21,81]
Sirolimus	mTORC1 inhibitor	mTORC1 (immunometabolism, autophagy)	Vienna single-center RCT (Redl 2024, n = 16, cutaneous): topical sirolimus ineffective; systemic 70% response (7/10) with median CSAMI of−7.0 (*p* = 0.018), 3 complete responders, and response sustained up to 2 years after 4-month course. Transplant registry: lower sarcoidosis incidence vs. calcineurin inhibitors. Case series: multisystem benefit in refractory disease.	Emerging—Phase 2 + Registry	[45,46,86]
XTMAB-16	Sarcoidosis-specific anti-TNF-α (chimeric mAb)	TNF-α	Phase 1b/2a Part A complete (NCT05890729, n = 39): clean safety, no DLTs; 4 mg/kg Q4W selected for advancement based on optimal balance of OCStaper, FVC stability (+0.5%), and low ADA incidence (14.3%). Planned Phase 2 efficacy trial (“XAtlas”) 2026. FDA + EMA orphan drug designations.	Emerging—Phase 1b/2a complete; Ph 2 planned	[77]
RCI (repository corticotropin)	Melanocortin agonist (porcine ACTH)	MC1R/MC3R	Phase 4 multicenter DB-RCT (Mirsaeidi 2023, n = 55): terminated earlyby COVID-19; descriptive trends favored RCI on novel Sarcoidosis Treatment Score (1.4 vs. 0.7 at week 24), steroid discontinuations, PFTs, andPROs. Validated STS endpoint for future trials. No new safety signals.	Hypothesis-generating (Phase 4 underpowered)	[91]
**TIER 3 · Phenotype-specific evidence or repurposed-agent signals**					
Sotatercept	Activin signaling inhibitor	Activin/BMP pathway	Recent report: improved lung function in sarcoidosis-associated pulmonary hypertension (SAPH). Parallels its established efficacy in PAH.	Emerging—Early-phase (SAPH)	[88]
Riociguat	Soluble guanylate cyclase stimulator	NO–sGC–cGMP pathway	1-year DB-RCT in SAPH (Baughman 2022, n = 16): modest 6MWD gain; no QoL effect.	Established (small RCT, modest effect)	[89]
PDE-4 inhibitors (apremilast and roflumilast)	Phosphodiesterase-4 inhibitor	cAMP →↓TNF-α, ↓IL-23	Retrospective single-center analysis (n = 51): steroid-sparing across cutaneous, pulmonary, and constitutional manifestations; favorable safety vs. systemic immunosuppression.	Emerging—Retrospective	[90]
Deucravacitinib	Selective TYK2 inhibitor (allosteric)	TYK2 → IL-12/IL-23/Type-I IFN	Case reports: resolution of cutaneous sarcoidosis. Mechanism specifically targets Th1/Th17.1 axis without JAK1/2/3 effects.	Experimental—Case reports	[42]
Abrocitinib	JAK1-selective inhibitor	JAK1 (Th2/Th17 receptors)	Case report: efficacy in tattoo-associated cutaneous sarcoidosis with low-dose corticosteroids.	Experimental—Case report	[41]
Ruxolitinib (topical)	JAK1/2 inhibitor (cream)	JAK1/2	Case reports: cutaneous sarcoidosis; localized non-systemic option with uncertain depth of granuloma penetration.	Experimental—Case reports	[82]
Ustekinumab	Anti-IL-12/IL-23 (p40 mAb)	Shared p40 subunit	Phase 2 (cutaneous + pulmonary): limited efficacy in pulmonary disease; case reports for refractory cutaneous and extrapulmonary involvement.	Emerging—Phase 2 (limited)	[83]
**TIER 4 · Phase 2 ongoing/preclinical or mechanism-driven**					
CHIT1 inhibitor (OATD-01)	Chitotriosidase inhibitor	CHIT1 (macrophage enzyme)	Phase 2 ongoing trial as potential first-line option. Strong preclinical rationale: CHIT1 is the most abundant macrophage-secreted enzyme in active disease and a long-standing biomarker.	Emerging—Phase 2 ongoing	[93]
Anti-TL1A mAb	TL1A neutralization	TL1A → PI3K/AKT	Preclinical: attenuates Th1/Th17 dysregulation and granuloma formation by interrupting PI3K/AKT signaling.	Experimental—Preclinical	[94]
βc-receptor antagonist	Common β-chain blocker	βc receptor (GM-CSF/IL-3/IL-5)	Preclinical: Reduces granuloma formation by simultaneously interrupting inflammatory signaling and aberrant macrophage lipid metabolism. More proximal node than ligand-only blockade.	Experimental—Preclinical	[52]
PI3K γ/δ inhibitors	Class I PI3K inhibitor	PI3K γ/δ → AKT	Preclinical: rescues Treg function and ameliorates pulmonary granulomas in murine models.	Experimental—Preclinical	[17]
**TIER 5 · Negative pivotal RCTs (mechanistically informative, not currently useful)**					
Namilumab *	Anti-GM-CSF (human mAb)	GM-CSF	RESOLVE-Lung Phase 2 RCT (van den Blink 2026): primary endpoint of rescue event reduction not met; all secondary endpoints (FVC, OCS taper, and KSQ) were negative. Sponsor discontinued sarcoidosis program.	Negative—Phase 2	[47,96]
Sarilumab *	Anti-IL-6R (human mAb)	IL-6R	DB-RCT withdrawal trial (Baker 2024, n = 15) in glucocorticoid-dependent sarcoidosis: failed as steroid-sparing therapy.	Negative—Phase 2	[45]
CMK389 *	Anti-IL-18 mAb	IL-18	Phase 2 proof-of-concept RCT (NCT04064242, n = 62): primary endpoint was negative—ΔFVC%pred −0.48 (CMK389) vs. +1.02 (placebo); Bayesian treatment difference of −1.49 (80% CrI −3.56 to 0.60), *p* = 0.18, favoring placebo. Safety unremarkable. Novartis discontinued the program (2024)	Negative—Phase 2	[95]

Abbreviations: ADA, anti-drug antibodies; BMP, bone morphogenetic protein; cAMP, cyclic adenosine monophosphate; CSAMI, Cutaneous Sarcoidosis Activity and Morphology Index; DLT, dose-limiting toxicity; FVC, forced vital capacity; HARS, histidyl-tRNA synthetase; IFN, interferon; IL, interleukin; JAK, Janus kinase; KSQ, King’s Sarcoidosis Questionnaire; mAb, monoclonal antibody; MC1R/MC3R, melanocortin receptors 1/3; MCID, minimal clinically important difference; mTORC1, mechanistic target of rapamycin complex 1; NRP2, neuropilin-2; OCS, oral corticosteroids; PI3K, phosphoinositide 3-kinase; PRO, patient-reported outcome; QoL, quality of life; RCT, randomized controlled trial; RWE, real-world evidence; SAPH, sarcoidosis-associated pulmonary hypertension; SC, subcutaneous; SGRQ, Saint George’s Respiratory Questionnaire; STAT, signal transducer and activator of transcription; STS, Sarcoidosis Treatment Score; Th, T helper; TL1A, TNF-like ligand 1A; TNF, tumor necrosis factor; TNFR, TNF receptor; Treg, regulatory T cell; TYK2, tyrosine kinase 2; 6MWD, 6min walk distance. † Phase 3 primary endpoint not met but with positive nominal secondary signals. * Negative pivotal RCT—currently not clinically deployable.

**Table 2:** Key completedclinical trials in sarcoidosis.

Trial/Study	Agent	Design	Key Findings	Status	Refs
**TIER A · Landmark positive RCTs affecting current practice**					
PREDMETH (NCT04314193)	Prednisone vs. Methotrexate	Multicenter non-inferiority RCT, treatment-naïve symptomatic pulmonary sarcoidosis, 24-week FVC primary endpoint	MTX met non-inferiority (5% FVC margin) despite slower early response. Less steroid-related morbidity; favorable PROs. Establishes MTX as first-line alternative.	Published 2025 (NEJM)	[59]
SARCORT (NCT03265405)	Prednisolone, 20 mg vs. 40 mg	Open-label RCT, n = 86, treatment-naïve pulmonary; composite primary (failure/progression/relapse)	Low-dose, 20 mg, non-inferior; relapse in ~43–46% in both arms, treatment failure in 14% vs. 9%, and FVC was equivalent. Halved cumulative steroid exposure.	Published 2023 (ERJ)	[65]
Baughman Infliximab Trial	Infliximab, 3 or 5 mg/kg	Phase 2 multicenter DB-RCT, n = 138, chronic pulmonary sarcoidosis; 24-week FVC primary, 52-week follow-up	Primary met: +2.5% FVC vs. placebo (*p* = 0.038). All major secondaries (SGRQ, 6MWT, dyspnea, and lupus pernio) were not significant. Posthoc: greater benefit in more severe diseases. No steroid taper protocolized.	Published 2006 (AJRCCM)	[70]
Judson Extrapulmonary Infliximab Trial	Infliximab vs. placebo	Phase 2 RCT, n = 92, extrapulmonary sarcoidosis refractory to chronic glucocorticoids; 24-week treatment, 24-month follow-up	Modest improvement at week 24 not maintained on 24-month follow-up. Suggests extrapulmonary patients may need long-term therapy. No steroid taper protocolized.	Published 2008 (ERJ)	[71]
Efzofitimod Phase 2 (NCT03824392)	Efzofitimod, 1/3/5 mg/kg IV Q4W vs. placebo	DB-RCT 2:1, n = 37, pulmonary sarcoidosis on stable corticosteroids	Well-tolerated, no immunogenicity. Dose of 5 mg/kg: steroid taper without relapse; +2.5% FVC; KSQ above MCID. Dose-response signal supported Ph 3 advancement.	Published 2023 (Chest)	[39]
**TIER B · Pivotal negative trials—clinically and mechanistically informative**					
EFZO-FIT (NCT05415137)	Efzofitimod, 3 or 5 mg/kg vs. placebo	Global Phase 3 DB-RCT, n = 268, pulmonary sarcoidosis on stable OCS; 48-week treatment with protocolized taper	Primary OCS-taper endpoint missed (5 mg/kg vs. placebo: −2.79 vs. −3.52 mg/day, *p* = 0.33). Positive nominal signals on KSQ-General, KSQ-Lung, and Fatigue Assessment Scale. Largest-ever sarcoidosis study. Due to positive secondary endpoints, big clinical significance.	Reported ERS Congress 2025	[80]
RESOLVE-Lung (NCT05314517)	Namilumab (anti-GM-CSF) vs. placebo	Phase 2 multinational DB-RCT, chronic active pulmonary sarcoidosis; rescue-event primary endpoint	Primary endpoint not met; all pre-specified secondary endpoints (FVC, OCS taper, and KSQ) were negative. Sponsor discontinued program. Tempers enthusiasm for GM-CSF blockade as monotherapy.	Published 2026 (ERJ)	[96]
Sarilumab Withdrawal Trial	Sarilumab (anti-IL-6R)	DB-RCT randomized withdrawal, n = 15, glucocorticoid-dependent sarcoidosis	Negative: failed to achieve steroid-sparing effect. Suggests IL-6 axis is downstream of, or redundant with, the dominant Th1/Th17.1 and TNF circuits in established disease.	Published 2024 (Rheumatology)	[87]
CMK389 (NCT04064242)	CMK389 (anti-IL-18)	Phase 2 quadruple-blind RCT, n = 62, chronic pulmonary sarcoidosis; 10 mg/kg IV q4w ×4; ΔFVC%pred at wk16 primary	Negative: ΔFVC%pred −0.48 vs. +1.02 placebo; Bayesian difference of −1.49 (80% CrI −3.56–0.60) favored placebo. Program discontinued in 2024.	Completed; negative; results posted	[95]
**TIER C · Phenotype-specific RCTs and mechanistic clinical studies**					
Sirolimus Cutaneous Trial (Vienna)	Sirolimus (topical and systemic)	Single-center randomized trial (Redl 2024), n = 16, persistent glucocorticoid-refractory cutaneous sarcoidosis	Topical was ineffective. Systemic: 70% (7/10) responded; median CSAMI of −7.0 (*p* = 0.018); 3 complete responders; response sustained up to 2 years after 4-month course. First RCT-level evidence for mTOR inhibition.	Published 2024 (Lancet Rheumatol)	[86]
Tofacitinib (NCT03793439)	Tofacitinib, 5 mg BID	Phase 1 open-label clinical-translational study, n = 10, cutaneous sarcoidosis with multi-organ assessment	Marked clinical improvement; transcriptomic confirmation of type-1 immunity suppression in lesional skin. First mechanism-of-action confirmation for JAK inhibition in human sarcoidosis.	Published 2022 (Nat Commun)	[20]
Bechman Meta-analysis	Biologic + targeted synthetic therapies	Systematic review and meta-analysis of biologic and targeted synthetic therapies in sarcoidosis (all phenotypes)	Significant FVC improvement and consistent steroid-sparing effect for anti-TNF therapy (chiefly infliximab). Limited evidence for most other targeted agents.	Published 2025 (Thorax)	[29]
RCI Phase 4 Trial	Repository corticotropin injection (Acthar Gel)	Phase 4 multicenter DB-RCT, pulmonary sarcoidosis, n = 55 (terminated early—COVID-19)	Underpowered for hypothesis testing. Descriptive trends favored RCI (STS at 24 wks: 1.4 vs. 0.7; more steroid discontinuations). Validated novel STS endpoint.	Published 2023 (Pulm Ther)	[91]
Methylprednisolone Pulse—Renal Sarcoidosis	IV methylprednisolone + oral pred vs. oral pred alone	RCT in sarcoidosis tubulointerstitial nephritis	Pulse therapy not superior to oral prednisone alone.	Published 2023 (NDT)	[56]

## Data Availability

No new data were created or analyzed in this study. Data sharing is not applicable to this article.

## Abbreviations

6MWD, 6min walk distance; 6MWT, 6min walk test; AASOG, Americas Association of Sarcoidosis and Other Granulomatous Disorders; ACE, angiotensin-converting enzyme; ACEIs, angiotensin-converting enzyme inhibitors; ACTH, adrenocorticotropic hormone; ADA, anti-drug antibodies; AKT, protein kinase B; ARBs, angiotensin receptor blockers; BAL, bronchoalveolar lavage; BALF, bronchoalveolar lavage fluid; BMP, bone morphogenetic protein; cAMP, cyclic adenosine monophosphate; CD, cluster of differentiation; cGMP, cyclic guanosine monophosphate; CHIT1, chitotriosidase; CNS, central nervous system; CrI, credible interval; CSAMI, Cutaneous Sarcoidosis Activity and Morphology Index; CXCR4, C-X-C chemokine receptor type 4; DLT, dose-limiting toxicity; DMARD, disease-modifying antirheumatic drug; DR3, death receptor 3; EMA, European Medicines Agency; FDA, US Food and Drug Administration; FDG-PET, fluorodeoxyglucose positron emission tomography; FOXP3, forkhead box P3; FVC, forced vital capacity; GM-CSF, granulocyte-macrophage colony-stimulating factor; HARS, histidyl-tRNA synthetase; HRCT, high-resolution computed tomography; IFN, interferon; IgG1, immunoglobulin G1; IL, interleukin; JAK, Janus kinase; KL-6, Krebs von den Lungen-6; KSQ, King’s Sarcoidosis Questionnaire; mAb, monoclonal antibody; MAPK, mitogen-activated protein kinase; MC1R/MC3R, melanocortin receptors 1/3; MCID, minimal clinically important difference; MCP-1, monocyte chemoattractant protein-1; MHC, major histocompatibility complex; MTX, methotrexate; mTORC1, mechanistic target of rapamycin complex 1; NF-κB, nuclear factor kappa B; NK, natural killer; NLRP3, NLR family pyrin domain containing 3; NO, nitric oxide; NRP2, neuropilin-2; OCS, oral corticosteroids; OX40, OX40/CD134 (tumor-necrosis-factor-receptor-superfamily costimulatory molecule); PAH, pulmonary arterial hypertension; PD-1, programmed cell death protein 1; PDE-4, phosphodiesterase-4; PFT, pulmonary function test; PI3K, phosphoinositide 3-kinase; PPP, pentose phosphate pathway; PRO, patient-reported outcome; QoL, quality of life; RAAS, renin-angiotensin-aldosterone system; RCI, repository corticotropin injection; RCT, randomized controlled trial; RORγt, RAR-related orphan receptor gamma t; RWE, real-world evidence; SAA, serum amyloid A; SAPH, sarcoidosis-associated pulmonary hypertension; SC, subcutaneous; sGC, soluble guanylate cyclase; SGLT2, sodium-glucose cotransporter 2; SGRQ, Saint George’s Respiratory Questionnaire; SHP2, Src-homology-2 domain-containing phosphatase 2; sIL-2R, soluble interleukin-2 receptor; STAT, signal transducer and activator of transcription; STS, Sarcoidosis Treatment Score; T-bet, T-box transcription factor TBX21; TCR, T-cell receptor; Th, T helper; TL1A, TNF-like ligand 1A; TNF, tumor necrosis factor; TNFR, tumor necrosis factor receptor; Treg, regulatory T cell; TYK2, tyrosine kinase 2; βc, common beta chain (shared GM-CSF/IL-3/IL-5 receptor subunit) WASOG, World Association for Sarcoidosis and Other Granulomatoses; ERS, European Respiratory Society.

## References

[1] Drent M., Crouser E.D., Grunewald J. (2021). Challenges of Sarcoidosis and Its Management. N. Engl. J. Med..

[2] Oh J., Kim S., Yim Y., Kim M.S., Hay S.I., Il Shin J., Yon D.K., GBD 2023 Global Chronic Respiratory Disease and Covid Collaborators (2026). Global, regional, and national burden of chronic respiratory diseases and impact of the COVID-19 pandemic, 1990–2023: A Global Burden of Disease study. Nat. Med..

[3] Papanikolaou I.C., Chytopoulos K., Kaitatzis D., Kostakis N., Bogiatzis A., Steiropoulos P., Drakopanagiotakis F. (2025). Phenotypes and Endotypes in Sarcoidosis: Unraveling Prognosis and Disease Course. Biomedicines.

[4] Belperio J.A., Shaikh F., Abtin F. (2021). Extrapulmonary sarcoidosis with a focus on cardiac, nervous system, and ocular involvement. EClinicalMedicine.

[5] Miedema J.R., Bonella F., Buschulte K., Culver D.A., Jeny F., Obi O.N., Rivera N.V., Spagnolo P., Veltkamp M., Wijsenbeek M. (2026). Sarcoidosis: A State-Of-The-Art Review. Eur. Respir. J..

[6] Fernández-Ramón R., Gaitán-Valdizán J.J., Martín-Varillas J.L. (2024). Clinical phenotypes of sarcoidosis using cluster analysis: A Spanish population-based cohort study. Clin. Exp. Rheumatol..

[7] Lhote R., Annesi-Maesano I., Nunes H. (2021). Clinical phenotypes of extrapulmonary sarcoidosis: An analysis of a French, multi-ethnic, multicenter cohort. Eur. Respir. J..

[8] Lin N.W., Arbet J., Mroz M.M. (2022). Clinical phenotyping in sarcoidosis using cluster analysis. Respir. Res..

[9] Raasing L., Vogels O.J.M., Veltkamp M., Grutters J.C. (2022). Infliximab decreases inflammatory activity but has no effect on small fiber neuropathy related symptoms in Dutch patients with sarcoidosis. Sarcoidosis Vasc. Diffus. Lung Dis..

[10] Zhang X., Zhuang Y., Xie Y. (2025). Global, regional and national burden of interstitial lung disease and pulmonary sarcoidosis, 1990–2021 and projection to 2040. Front. Med..

[11] Rivera N.V., Israël-Biet D. (2025). Sarcoidosis in the Genomic Era: From Genetic Drivers to Tailored Therapies. Curr. Allergy Asthma Rep..

[12] Miedema J.R., de Jong L.J., van Uden D. (2024). Circulating T cells in sarcoidosis have an aberrantly activated phenotype that correlates with disease outcome. J. Autoimmun..

[13] Nakamizo S., Sugiura Y., Ishida Y. (2023). Activation of the pentose phosphate pathway in macrophages is crucial for granuloma formation in sarcoidosis. J. Clin. Investig..

[14] Zhao Y., Zhang X., Dong L. (2026). The interplay between M2-like macrophages and Th17.1 cells promotes the progression of early-stage sarcoidosis. Cell Commun. Signal..

[15] Kusaka K., Miyazaki Y., Nakayamada S. (2025). The abundance of regulatory T cell subsets is associated with the clinical outcomes of sarcoidosis. Mod. Rheumatol..

[16] Patterson K.C., Miller W.T., Hancock W.W., Akimova T. (2023). FOXP3+ regulatory T cells are associated with the severity and prognosis of sarcoidosis. Front. Immunol..

[17] Zhang X., Dai Q., Shan J. (2023). Inhibition of phosphoinositide-3 kinases γ/δ ameliorates pulmonary granuloma by rescuing Treg function in a sarcoidosis model. Exp. Ther. Med..

[18] Adouli J., Fried A., Swier R., Ghio A., Petrache I., Tilley S. (2023). Cellular Recycling Gone Wrong: The Role of Dysregulated Autophagy and Hyperactive mTORC1 in the Pathogenesis of Sarcoidosis. Sarcoidosis Vasc. Diffus. Lung Dis..

[19] Pizzini A., Bacher H., Aichner M. (2021). High expression of mTOR signaling in granulomatous lesions is not predictive for the clinical course of sarcoidosis. Respir. Med..

[20] Damsky W., Wang A., Kim D.J. (2022). Inhibition of type 1 immunity with tofacitinib is associated with marked improvement in longstanding sarcoidosis. Nat. Commun..

[21] Quaggetto M., Ben Salem T., Haroche J. (2025). Janus kinase inhibitors in pulmonary and extra-pulmonary sarcoidosis: A case series and a systematic review of the literature. Sarcoidosis Vasc. Diffus. Lung Dis..

[22] Miedema J.R., de Jong L.J., Kahlmann V. (2024). Increased proportions of circulating PD-1+ CD4+ memory T cells and PD-1+ regulatory T cells associate with good response to prednisone in pulmonary sarcoidosis. Respir. Res..

[23] Saw P.E., Song E. (2025). The ‘inflammazone’ in chronic inflammatory diseases: Psoriasis and sarcoidosis. Trends Immunol..

[24] Kvacskay P., El Jammal T., Lorenz H.M., Pacheco Y., Calender A. (2024). Whole exome sequencing of a German sarcoidosis family with four affected and one spontaneous remission case. Rheumatology.

[25] Zlatar L., Knopf J., Singh J. (2024). Neutrophil extracellular traps characterize caseating granulomas. Cell Death Dis..

[26] Cattran A., Culver D.A. (2025). Treatment of Sarcoidosis Over the Next Decade. Semin. Respir. Crit. Care Med..

[27] Wells A.U., Lower E.E., Baughman R.P. (2025). A paradigm shift in corticosteroid therapy for sarcoidosis: A World Association of Sarcoidosis and Other Granulomatous Disorders Position Paper, endorsed by the Americas Association of Sarcoidosis and Other Granulomatous Disorders. Lancet Respir. Med..

[28] Baughman R.P., Grutters J.C., Lower E.E. (2025). Pulmonary sarcoidosis clinical trial end-points: A Delphi study. Eur. Respir. J..

[29] Bechman K., Biddle K., Miracle A. (2025). Systematic review and meta-analysis of the efficacy of biologic and targeted synthetic therapies in sarcoidosis. Thorax.

[30] Rossides M., Arkema E.V. (2025). Sarcoidosis Treatment Patterns in the United States: The Need For Real-World Evidence to Inform Future Practice. Chest.

[31] Sangani R., Bosch N.A., Govender P. (2025). Sarcoidosis Treatment Patterns in the United States: 2016–2022. Chest.

[32] Judson M.A., Ouedraogo W.O., Fish K.M. (2024). Factors Associated with Corticosteroid Adherence in Sarcoidosis. Lung.

[33] Bechman K., Galloway J., Birring S.S. (2026). Towards a new treatment era in sarcoidosis. Lancet Respir. Med..

[34] Kullberg S., Rivera N.V., Grunewald J., Eklund A. (2021). Effects of infliximab on lung and circulating natural killer cells, CD56+ T cells and B cells in sarcoidosis. BMJ Open Respir. Res..

[35] Padhi A., Eklund A., Malmeström C. (2025). Associations of peripheral blood lymphopenia to disease course, treatment and TNF-α in sarcoidosis. Respir. Res..

[36] Ziegenhagen M.W., Benner U.K., Zissel G., Zabel P., Schlaak M., Muller-Quernheim J. (1997). Sarcoidosis: TNF-alpha release from alveolar macrophages and serum level of sIL-2R are prognostic markers. Am. J. Respir. Crit. Care Med..

[37] Xu D., Tao X., Fan Y., Teng Y. (2025). Sarcoidosis: Molecular mechanisms and therapeutic strategies. Mol. Biomed..

[38] Baughman R.P., Niranjan V., Walker G. (2023). Efzofitimod: A novel anti-inflammatory agent for sarcoidosis. Sarcoidosis Vasc. Diffus. Lung Dis..

[39] Culver D.A., Aryal S., Barney J. (2023). Efzofitimod for the Treatment of Pulmonary Sarcoidosis. Chest.

[40] Nangle L.A., Xu Z., Siefker D. (2025). A human histidyl-tRNA synthetase splice variant therapeutic targets NRP2 to resolve lung inflammation and fibrosis. Sci. Transl. Med..

[41] Geng Q., Xu J. (2025). Abrocitinib combined with low-dose corticosteroids in the management of tattoo-related cutaneous sarcoidosis: A case report. J. Dermatol. Treat..

[42] Herrera H.O., Sanchez C., Minor G., Fulchiero G.J. (2025). Successful treatment of cutaneous sarcoidosis with deucravacitinib, a selective TYK2 inhibitor. JAAD Case Rep..

[43] Schreiber T., Falk-Paulsen M., Kuiper J. (2021). IL23R on myeloid cells is involved in murine pulmonary granuloma formation. Exp. Lung Res..

[44] Zhang H., Jiang D., Zhu L. (2023). Imbalanced distribution of regulatory T cells and Th17.1 cells in the peripheral blood and BALF of sarcoidosis patients: Relationship to disease activity and the fibrotic radiographic phenotype. Front. Immunol..

[45] Baker M.C., Vágó E., Liu Y. (2022). Sarcoidosis incidence after mTOR inhibitor treatment. Semin. Arthritis Rheum..

[46] McGuire L., Brown R., Asimaki A. (2025). Use of Sirolimus, an mTOR Inhibitor, to Treat Sarcoidosis in Multiple Systems. J. Cardiovasc. Transl. Res..

[47] van den Blink B., Petit C.M., Dansky H.M. (2025). Design of RESOLVE Lung, a multinational Phase 2, randomized, placebo-controlled trial of the anti-GM-CSF monoclonal antibody namilumab in patients with chronic pulmonary sarcoidosis. Contemp. Clin. Trials.

[48] Fares J., El Fadel O., Zhao J. (2025). Mortality and Health Outcomes Among Patients With Sarcoidosis Treated With Angiotensin-Converting Enzyme Inhibitors and Angiotensin Receptor Blockers. Chest.

[49] Sati S., Huang J., Kersh A.E. (2024). Recruitment of CXCR4+ type 1 innate lymphoid cells distinguishes sarcoidosis from other skin granulomatous diseases. J. Clin. Investig..

[50] Celada S.I., Lim C.X., Carisey A.F. (2023). SHP2 promotes sarcoidosis severity by inhibiting SKP2-targeted ubiquitination of TBET in CD8+ T cells. Sci. Transl. Med..

[51] Kumari R., Chakraborty S., Jain R. (2021). Inhibiting OX40 Restores Regulatory T-Cell Function and Suppresses Inflammation in Pulmonary Sarcoidosis. Chest.

[52] Wang H., Tumes D.J., Keam S. (2025). βc receptor antagonism mitigates sarcoidosis granuloma formation by targeting inflammatory signals; aberrant lipid metabolism. Front. Immunol..

[53] Talreja J., Peng C., Zhang K., Samavati L. (2025). Novel Sarcoidosis Epitope Augments MHCII, CD80, and CD86 Expression and Promotes B-Cell Differentiation and IgG Production. Am. J. Respir. Cell Mol. Biol..

[54] Miyashita K., Hashimoto K., Maeda S., Suda T. (2025). Oral Corticosteroid Use and Its Associated Complications in Patients With Sarcoidosis: A Nationwide Claims Study From Japan. Cureus.

[55] Baughman R.P., Valeyre D., Korsten P., Mathioudakis A.G., Wuyts W.A., Wells A., Rottoli P., Nunes H., Lower E.E., Judson M.A. (2021). ERS clinical practice guidelines on treatment of sarcoidosis. Eur. Respir. J..

[56] Mahevas M., Audard V., Rousseau A. (2023). Efficacy and safety of methylprednisolone pulse followed by oral prednisone vs. oral prednisone alone in sarcoidosis tubulointerstitial nephritis: A randomized, open-label, controlled clinical trial. Nephrol. Dial. Transplant..

[57] Gottlieb J.E., Israel H.L., Steiner R.M. (1997). Outcome in sarcoidosis. The relationship of relapse to corticosteroid therapy. Chest.

[58] Selroos O., Brattsand R. (2024). Inhaled budesonide and pulmonary sarcoidosis revisited. Sarcoidosis Vasc. Diffus. Lung Dis..

[59] Kahlmann V., Janssen Bonás M., Moor C.C. (2025). First-Line Treatment of Pulmonary Sarcoidosis with Prednisone or Methotrexate. N. Engl. J. Med..

[60] Janssen Bonás M., Sundaresan J., Keijsers R.G.M. (2023). Methotrexate Polyglutamate Concentrations as a Possible Predictive Marker for Effectiveness of Methotrexate Therapy in Patients with Sarcoidosis: A Pilot Study. Lung.

[61] Leclercq M., Sève P., Biard L. (2024). Methotrexate versus conventional disease-modifying antirheumatic drugs in the treatment of non-anterior sarcoidosis-associated uveitis. Br. J. Ophthalmol..

[62] Jacquot R., Sève P., Mulier G. (2025). Corticosteroids with or without Conventional Disease-Modifying Antirheumatic Drug as First-Line Therapy in Nonanterior Sarcoidosis Uveitis. Ophthalmology.

[63] Vermeer B., Veltkamp M., Raasing L.R.M., Wind A.E., Vorselaars A.D.M. (2024). Hydroxychloroquine monotherapy in sarcoidosis: Indications, efficacy, and side effects. Sarcoidosis Vasc. Diffus. Lung Dis..

[64] Bitoun S. (2016). Treatment of neurosarcoidosis. A comparative study of methotrexate and mycophenolate mofetil. Neurology.

[65] Dhooria S., Sehgal I.S., Agarwal R. (2023). High-dose (40 mg) versus low-dose (20 mg) prednisolone for treating sarcoidosis: A randomised trial (SARCORT trial). Eur. Respir. J..

[66] Sawahata M., Kimura H., Hattori T. (2025). Clinical characteristics of patients with pulmonary sarcoidosis treated with systemic steroids in Japan. Front. Med..

[67] Onuora S. (2025). Methotrexate as first-line therapy for pulmonary sarcoidosis. Nat. Rev. Rheumatol..

[68] Baughman R.P., Lower E.E. (2025). Methotrexate as Initial Therapy for Symptomatic Pulmonary Sarcoidosis?. N. Engl. J. Med..

[69] Alipour P., Nery P.B., Beanlands R.S. (2025). Durable remission of cardiac sarcoidosis following discontinuation of methotrexate: A prospective cohort study. Respir. Med..

[70] Baughman R.P., Drent M., Kavuru M., Judson M.A., Costabel U., du Bois R., Albera C., Brutsche M., Davis G., Donohue J.F. (2006). Infliximab therapy in patients with chronic sarcoidosis and pulmonary involvement. Am. J. Respir. Crit. Care Med..

[71] Judson M.A., Baughman R.P., Costabel U., Flavin S., Lo K.H., Kavuru M.S., Drent M., Centocor T.S.I. (2008). Efficacy of infliximab in extrapulmonary sarcoidosis: Results from a randomised trial. Eur. Respir. J..

[72] Sakkat A., Cox G., Khalidi N. (2022). Infliximab therapy in refractory sarcoidosis: A multicenter real-world analysis. Respir. Res..

[73] Sweis J.J.G., Sweis N.W.G., Ascoli C. (2022). Adalimumab in the treatment of cardiac sarcoidosis: Single center case series and narrative literature review. Respir. Med. Case Rep..

[74] Crommelin H.A., van der Burg L.M., Vorselaars A.D., Drent M., van Moorsel C.H., Rijkers G.T., Deneer V.H., Grutters J.C. (2016). Efficacy of adalimumab in sarcoidosis patients who developed intolerance to infliximab. Respir. Med..

[75] Otten B., Weinberg D., Gregoski M.J., James W.E. (2025). Management of anti-drug antibodies against TNF-inhibitors in sarcoidosis patients. Respir. Med..

[76] Yee A.M.F. (2023). Durable medication-free remission of sarcoidosis following discontinuation of anti-tumor necrosis factor-α therapy. Respir. Med..

[77] Offman E., Singh N., Julian M.W. (2023). Leveraging in vitro and pharmacokinetic models to support bench to bedside investigation of XTMAB-16 as a novel pulmonary sarcoidosis treatment. Front. Pharmacol..

[78] Walker G., Adams R., Guy L. (2023). Exposure-response analyses of efzofitimod in patients with pulmonary sarcoidosis. Front. Pharmacol..

[79] Obi O.N., Baughman R.P., Crouser E.D. (2025). Therapeutic doses of efzofitimod demonstrate efficacy in pulmonary sarcoidosis. ERJ Open Res..

[80] Culver D., Bonella F., Carey L., Ramesh P., Chandrasekaran A., Kinnersley N., Niranjan V., Baughman R. EFZO-FIT: The Largest Ever Interventional Trial in Pulmonary Sarcoidosis. Proceedings of the ERS Congress 2025.

[81] Leffers H.C.B., Baslund B., Lindhardsen J., Krintel S.B., Graudal N. (2024). Abatacept and tofacitinib in refractory sarcoidosis: Drug survival, safety, and treatment response. Clin. Exp. Rheumatol..

[82] Smith J.S., Woodbury M.J., Merola J.F. (2023). Ruxolitinib cream for the treatment of cutaneous sarcoidosis. JAAD Case Rep..

[83] Judson M.A., Baughman R.P., Costabel U., Drent M., Gibson K.F., Raghu G., Shigemitsu H., Barney J.B., Culver D.A., Hamzeh N.Y. (2014). Safety and efficacy of ustekinumab or golimumab in patients with chronic sarcoidosis. Eur. Respir. J..

[84] Gad M.M., Bazarbashi N., Kaur M., Gupta A. (2019). Sarcoid-like Phenomenon—Ustekinumab induced granulomatous reaction mimicking diffuse metastatic disease: A case report and review of the literature. J. Med. Case Rep..

[85] Kobak S., Semiz H. (2020). Ustekinumab-induced Sarcoidosis in a Patient with Psoriatic Arthritis. Curr. Drug Saf..

[86] Redl A., Doberer K., Unterluggauer L., Kleissl L., Krall C., Mayerhofer C., Reininger B., Stary V., Zila N., Weninger W. (2024). Efficacy and safety of mTOR inhibition in cutaneous sarcoidosis: A single-centre trial. Lancet Rheumatol..

[87] Baker M.C., Horomanski A., Wang Y. (2024). A double-blind, placebo-controlled, randomized withdrawal trial of sarilumab for the treatment of glucocorticoid-dependent sarcoidosis. Rheumatology.

[88] Poor H.D., Eisenberg E., Saini S., Hannah-Clark S., Zhang J., Lee A.G., Serrao G., Powell C., Ventetuolo C.E., Padilla M. (2026). Association Between Sotatercept and Improved Lung Function in Sarcoidosis-Associated Pulmonary Hypertension. Chest.

[89] Baughman R.P., Shlobin O.A., Gupta R. (2022). Riociguat for Sarcoidosis-Associated Pulmonary Hypertension: Results of a 1-Year Double-Blind, Placebo-Controlled Trial. Chest.

[90] Feineis M.E., Terschluse C., Jouanjan L. (2025). PDE-4 Inhibition in Sarcoidosis Patients: A Retrospective Single-Center Analysis of 51 Patients. Pharmaceuticals.

[91] Mirsaeidi M., Baughman R.P., Sahoo D., Tarau E. (2023). Results From a Phase 4, Multicenter, Randomized, Double-Blind, Placebo-Controlled Study of Repository Corticotropin Injection for the Treatment of Pulmonary Sarcoidosis. Pulm. Ther..

[92] Alam A.B.M.N., Gill N., Han I., Nagasaka R., Hu W., Shamsuddin L. (2026). Repurposing sodium glucose cotransporter-2 (SGLT-2) inhibitors in sarcoidosis: A potential strategy for reducing mortality. Heart Lung.

[93] Dymek B., Sklepkiewicz P., Mlacki M. (2022). Pharmacological Inhibition of Chitotriosidase (CHIT1) as a Novel Therapeutic Approach for Sarcoidosis. J. Inflamm. Res..

[94] Ma C., Huang J., Zheng Y. (2024). Anti-TL1A monoclonal antibody modulates the dysregulation of Th1/Th17 cells and attenuates granuloma formation in sarcoidosis by inhibiting the PI3K/AKT signaling pathway. Int. Immunopharmacol..

[95] A Study of Efficacy, Safety and Tolerability of CMK389 in Chronic Pulmonary Sarcoidosis (NCT04064242). ClinicalTrials.gov Results, Posted 2025. NCT04064242.

[96] Van Den Blink B., Birring S.S., Mogulkoc N., Atis S.N., Gupta R., Guiot J., Hart S.P., Carmona Porquera E.M., Koeller H.B., Arocho N. (2026). Safety and efficacy of namilumab for the treatment of chronic pulmonary sarcoidosis (RESOLVE-Lung): A randomized, double-blinded, multicenter, Phase 2 study. Eur. Respir. J..

[97] Bernaudin J.F., Jeny F., Valeyre D. (2026). Considering molecular pharmacology for sarcoidosis treatment. Lancet Respir. Med..

[98] Kraaijvanger R., Janssen Bonás M., Paspali I. (2025). Targeted proteomics in extracellular vesicles identifies biomarkers predictive for therapeutic response in sarcoidosis. ERJ Open Res..

[99] Kraaijvanger R., Janssen Bonás M., Grutters J.C. (2024). Decreased serpin C1 in extracellular vesicles predicts response to methotrexate treatment in patients with pulmonary sarcoidosis. Respir. Res..

[100] Pascual M.B., Zapata-Huizi J.J., Ramos D. (2025). Biomarkers in Sarcoidosis: From Traditional Markers to Precision Medicine. Semin. Respir. Crit. Care Med..

[101] Paul P., Dasgupta S., RoyChowdhury S. (2025). Proteomics in interstitial lung disease: New insights into pathogenesis, diagnosis and treatment. Respir. Med..

[102] Dasgupta S., Choudhury P., Patidar S. (2025). Integrative analysis of transcriptome and metabolome profiles reveals immune-metabolic alterations in pulmonary sarcoidosis. Metabolomics.

[103] Zhu S., Hao Z., Chen Q. (2024). Casual effects of telomere length on sarcoidosis: A bidirectional Mendelian randomization analysis. Front. Med..

[104] Vithalkar M.P., Sandra K.S., Bharath H.B., Krishnaprasad B., Fayaz S.M., Sathyanarayana B., Nayak Y. (2025). Network Pharmacology-driven therapeutic interventions for Interstitial Lung Diseases using Traditional medicines: A Narrative Review. Int. Immunopharmacol..

[105] Pianigiani T., Perea B., Dilroba A. (2026). Uncovering Sex and Gender Differences in Sarcoidosis: A Systematic Review of Current Evidence. J. Pers. Med..

[106] Obi O.N., Arkema E.V., Cozier Y.C. (2026). Patterns and trends in sarcoidosis: An epidemiological perspective. Curr. Opin. Immunol..

[107] d’Alessandro M., Gangi S., Paggi I., Soccio P., Bergantini L., Pianigiani T., Montuori G., Moriondo G., Natalello G., Marrucci S. (2024). Diagnostic Performance of CLEIA Versus FEIA for KL-6 Peripheral and Alveolar Concentrations in Fibrotic Interstitial Lung Diseases: A Multicentre Study. J. Clin. Lab. Anal..

[108] Zuo L., Zhang W., Wang Y., Qi X. (2024). Diagnostic Value of Serum KL-6 in Interstitial Lung Diseases. Int. J. Gen. Med..

[109] Sharp M., Psoter K.J., Mustafa A.M. (2024). Pulmonary sarcoidosis: Differences in lung function change over time. Thorax.

